# Genome signature-based dissection of human gut metagenomes to extract subliminal viral sequences

**DOI:** 10.1038/ncomms3420

**Published:** 2013-09-16

**Authors:** Lesley A. Ogilvie, Lucas D. Bowler, Jonathan Caplin, Cinzia Dedi, David Diston, Elizabeth Cheek, Huw Taylor, James E. Ebdon, Brian V. Jones

**Affiliations:** 1 Centre for Biomedical and Health Science Research, School of Pharmacy and Biomolecular Sciences, University of Brighton, Brighton BN2 4GJ, UK; 2 School of Environment and Technology, University of Brighton, Brighton BN2 4GJ, UK; 3 School of Computing, Engineering and Mathematics, University of Brighton, Brighton BN2 4GJ, UK; 4 Present address: Mikrobiologische and Biotechnologische Risiken Bundesamt für Gesundheit BAG, 3003 Bern, Switzerland

## Abstract

Bacterial viruses (bacteriophages) have a key role in shaping the development and functional outputs of host microbiomes. Although metagenomic approaches have greatly expanded our understanding of the prokaryotic virosphere, additional tools are required for the phage-oriented dissection of metagenomic data sets, and host-range affiliation of recovered sequences. Here we demonstrate the application of a genome signature-based approach to interrogate conventional whole-community metagenomes and access subliminal, phylogenetically targeted, phage sequences present within. We describe a portion of the biological dark matter extant in the human gut virome, and bring to light a population of potentially gut-specific *Bacteroidales*-like phage, poorly represented in existing virus like particle-derived viral metagenomes. These predominantly temperate phage were shown to encode functions of direct relevance to human health in the form of antibiotic resistance genes, and provided evidence for the existence of putative ‘viral-enterotypes’ among this fraction of the human gut virome.

## Abstract (web summary)

Bacteriophages have a significant impact on microbial ecosystems, but additional tools are needed to assess viral communities. Ogilvie *et al.* present a new strategy to extract viral sequences from metagenomic data sets, and present new insights on their function in the gut ecosystem.

## Graphical abstract

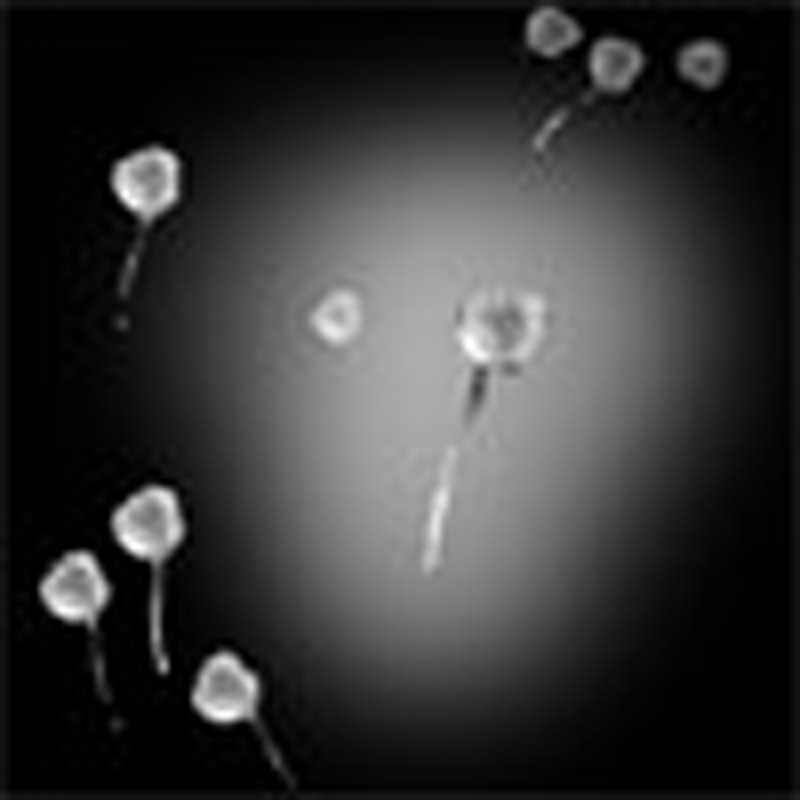

Viruses are the most abundant infectious agents on the planet, and collectively constitute a highly diverse and largely unexplored gene-space, which accounts for much of the ‘biological dark matter’ in Earth’s biosphere^1^,^2^,^3^. Bacterial viruses (bacteriophage or phage) are considered the most numerous viral entities, and through their effects on host bacteria, phage can influence processes ranging from global geochemical cycles to bacterial virulence and pathogenesis^1^,^2^,^3^,^4^,^5^. The study of this expansive family of viruses continues to underpin many fundamental insights into microbial physiology and evolution, with the interplay of bacteria and phage now studied at scales ranging from the individual components of single-phage species, to community-level surveys of viral assemblages and their impacts on host microbial ecosystems.

The development of metagenomic tools for analysis of phage populations constitutes a major advance in this regard, which is poised to deliver unprecedented insight into the prokaryotic virosphere. This powerful culture-independent approach overcomes many limitations of traditional methods for phage isolation and characterization, ultimately promising almost unrestricted access to the genetic content of host microbiomes and their attendant viral collectives^3^,^6^,^7^,^8^,^9^,^10^,^11^. Application of these techniques to the study of microbial viromes has already provided major insights into a number of phage communities, including those associated with microbial ecosystems that develop in or on the human body^7^,^11^,^12^.

In particular, the retinue of phage associated with the human gut microbiome is now increasingly recognized as an important facet of this ecosystem, which may significantly influence its impact on human health^3^,^5^,^13^,^14^,^15^,^16^. Gut-associated phage have already been shown to encode genes that confer production of toxins, virulence factors or antibiotic resistance upon host bacteria^5^,^17^,^18^, and have the potential to modulate community structure and metabolic output through elimination of host species or introduction of new traits^1^,^16^,^19^. Furthermore, virome composition also appears to be altered in disease states, which has given rise to the hypothesis that the human gut virome may have a role in the pathogenesis of disorders associated with perturbation of the gut ecosystem^14^. Phage also hold considerable biotechnological and pharmaceutical potential, with the gut virome now a viable target for bio-prospecting and the development of novel therapeutic or diagnostic tools^3^,^13^.

However, current strategies for generating viral metagenomes are not without limitations, and are typically based on analysis of nucleic acids derived from purified virus like particles (VLPs)^3^,^7^,^11^,^20^. As such, these approaches are targeted towards analysis of free-phage particles present at the time of sampling, which restricts access to the quiescent virome fraction and obscures host-range information^8^. VLP-based approaches will also poorly represent phage not efficiently recovered during virion purification stages, and typically rely on subsequent amplification of extracted viral DNA before sequencing, which can also exclude some phage types^3^,^7^,^11^,^20^. Although these caveats do not undermine the overall utility of the VLP approach (which retains a clear advantage in accessing actively replicating phage), much scope remains to develop complementary strategies to access and analyse microbial viromes.

In this context, it is notable that conventional metagenomic data sets, derived from total community DNA, have been found to contain significant fractions of phage sequence data, and in the case of the gut microbiome, this has been estimated to be up to 17% of microbial DNA recovered from stool samples^7^,^11^,^21^. Owing to the focus on acquisition of chromosomal sequences and an independence from VLP extracts, these data sets are likely to capture prophage not readily accessed by VLP-based surveys^8^, and will by default also contain much genetic material from phage–host species or closely related organisms. The latter should facilitate inference of host-range and permit a more in-depth analysis of the local ecological landscape populated by recovered phage, and together with the former stands to provide an alternative and novel perspective on the gut virome. Therefore, whole-community metagenomes may constitute valuable resources for the analysis of phage communities, and in conjunction with VLP-derived data sets, provide a more complete understanding of phage concurrent with the human gut and other ecosystems^8^.

Nevertheless, the resolution and host-range affiliation of phage fragments present in conventional metagenomes remains challenging, with particular problems arising from the paucity of well-characterized phage reference genomes with established host ranges, a lack of universally conserved and robust phylogenetic anchors in phage genomes (akin to bacterial 16S rRNA genes), as well as the mosaic nature of phage genomes, and the fragmentary nature of metagenomic data sets^8^,^13^. These factors, in conjunction with the potential value of standard metagenomes for virome analysis, highlight the need to develop robust approaches for phage-oriented dissection of these repositories, and host-range affiliation of recovered phage sequences.

Here we demonstrate the application of a genome signature-based approach for retrieval of subliminal, phylogenetically targeted phage sequences present within conventional gut microbial metagenomes. Application of this strategy permitted the identification of a subset of gut-specific *Bacteroidales*-like phage sequences poorly represented in existing VLP-derived viral metagenomes. These phage sequences were shown to encode functions of direct relevance to human health, and provided new insights into the structure and composition of the human gut virome.

## Results

### Genome signature-based recovery of ‘*Bacteroidales*-like’ phage

Members of the *Bacteroidales*, and in particular the genus *Bacteroides,* are abundant and important constituents of the human gut microbiome for which few complete phage genomes are available, with this region of the gut virome believed to remain largely uncharted^13^. To more fully explore this novel phage gene-space, we utilized *Bacteroidales* phage sequences as ‘drivers’ to interrogate 139 human gut metagenomes based on tetranucleotide usage profiles (TUPs) and functional profiles of contigs (Table 1, Supplementary Figs S1–S3, Supplementary Table S1).

**Figure 1:**
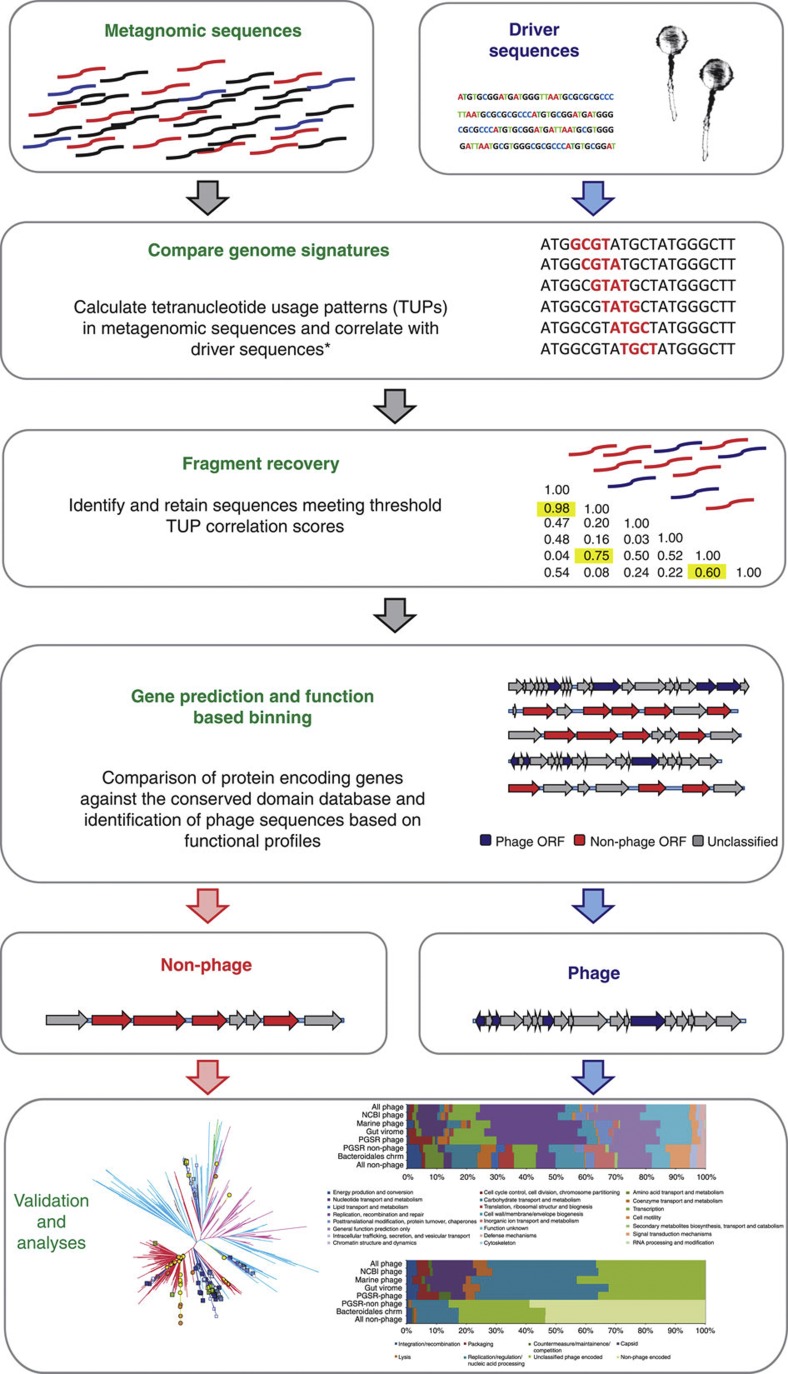
Overview of the PGSR approach. TUPs of all large fragments (10 kb or over) from 139 human gut metagenomes were calculated, and compared with those of phage genome sequences used as drivers. All metagenomic fragments producing tetranucleotide correlation values of 0.6 or over to any driver sequence were retained, and subjected to functional profiling to resolve phage and non-phage sequences captured. See Table 1 and Supplementary Figs S1–S3 for details of driver sequences. See Supplementary Table S1 for details of human gut metagenomes utilized. *Tetranucleotide usage patterns and correlations were calculated using TETRA 1.0 (ref. 46).

This strategy takes advantage of similarities in global nucleotide usage patterns, or the genome signature, arising between phage infecting the same or related host bacterial species^22^,^23^,^24^. We exploit this phenomenon to identify contigs related to *Bacteroidales* phage driver sequences in assembled gut metagenomes, and subsequent function-based binning to resolve phage fragments recovered in this process (Fig. 1). We refer to this strategy as phage genome signature-based recovery (PGSR), and denote sequences obtained in this way with the PGSR prefix.

Interrogation of all large contigs (10 kb and over) from human gut metagenomes (Supplementary Table S1) recovered 408 metagenomic fragments with TUPs similar to *Bacteroidales* phage drivers. Eighty five fragments were categorized as phage based on functional profiling, and the remainder classified as non-phage (presumed chromosomal, *n*=320), or could not be categorized (*n*=3) (Supplementary Data 1). The proportion of sequences categorized as phage within the total pool of 408 sequences recovered by PGSR (20.83%; 85/408) is congruent with recent studies estimating that up to 17% of total metagenomic DNA derived from stool samples may be viral in origin^7^,^11^,^21^. Of the PGSR sequences classified as phage, sizes ranged from 10–63.7 kb, with 16 sequences over 30 kb in length (Supplementary Data 1). This size range is consistent with that of available *Bacteroides* phage genomes used as drivers, and phage types known to be prominent within the human gut virome (particularly members of the *Siphoviridae* family)^11^, pointing to the recovery of near full-length or complete phage genomes.

### Recovery of contiguous phage genome fragments

**Figure 2:**
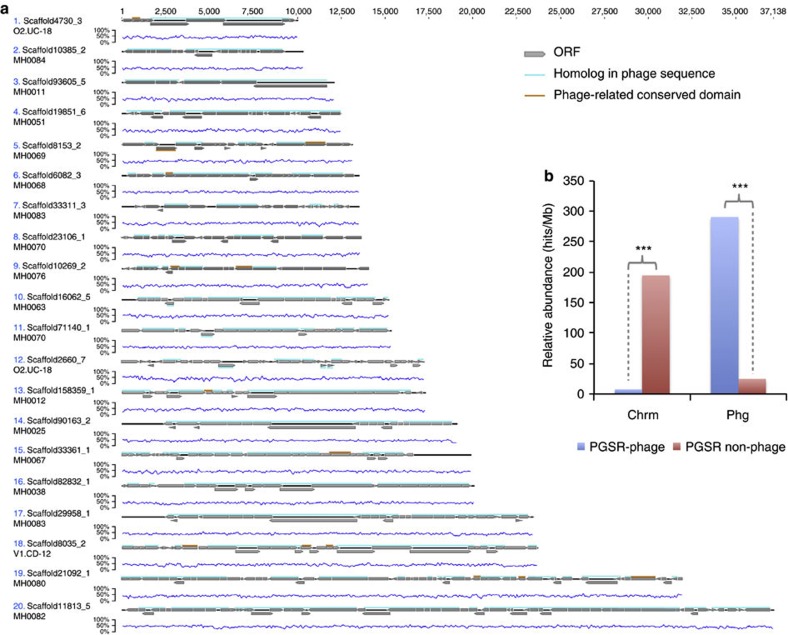
Analysis of chromosomal contamination in PGSR phage sequences. Owing to the dominance of chromosomal sequences in the metagenomic data sets analysed and the likelihood that many PGSR phage represent integrated prophage, PGSR phage were examined for the presence of terminal chromosomal regions. (**a**) Physical maps of 20 randomly selected PGSR phage sequences indicating ORFs with homologues in other phage sequences. Graphs associated with each phage sequence show % G+C across the sequence. ORF homologues in phage data sets were identified based on tBlastn searches (1e^−3^ or lower) of 711 complete or partial phage genomes, and all contigs assembled from human gut viral metagenomes^11^. ORFs highlighted in cyan have homologues in phage genomes. ORFs highlighted in red generated no valid hits to phage sequences but encode conserved domains with phage-related functions (for example, capsid, integrase and recombination/replication). (**b**) Relative abundance of ORFs homologous to those encoded by PGSR phage and PGSR non-phage contigs, in phage sequences (711 phage genomes, PGSR phage sequences and assemblies of human gut viromes) and chromosomes (1,821 chromosomes and all PGSR non-phage) expressed as hits per Mb DNA (valid hits=minimum 35% identity over 30 aa or more, 1e^−5^ or lower). ***P≤0.001 (*χ*^2^-test). Data sets and sequences utilized are described in Supplementary Table S1, Supplementary Data 3–6).

Owing to the dominance of chromosomal sequences in the metagenomic data sets examined, and the corollary that many PGSR phage fragments could therefore be chimeras corresponding to chromosome–prophage junctions, we also assessed the fidelity of the PGSR approach in this regard. Initially, 20 PGSR phage sequences were randomly selected, annotated and each open reading frame (ORF) evaluated in terms of their association with phage genomes (Fig. 2a). The majority of sequences examined were shown to encode a clear and consistent phage-related signal across their entire length, with gene architectures and organization commensurate with driver phage genomes (Supplementary Fig. S3). A potential exception of note being sequence no. 9, which exhibited a terminal region devoid of phage-related ORFs, indicating the possible presence of terminal chromosomal sequences (Fig. 2a).

In an extension of this analysis, all protein encoding genes from all PGSR phage and PGSR non-phage contigs were used to search an extensive collection of phage and chromosomal sequences (Fig. 2b). Results of these searches were used to calculate the relative abundance of homologous ORFs from PGSR sequences in phage genomes and chromosomes (Fig. 2b). This demonstrated that the vast majority of genes from PGSR phage sequences were well represented in other phage genomes and phage data sets, but exhibited significantly lower relative abundance in chromosomal sequences analysed (Fig. 2b). For PGSR non-phage sequences, which are presumed to be chromosomal in origin, the converse was true with high levels of representation in chromosomal sequences but a low relative abundance in phage sequences (Fig. 2b). Taken together, these analyses demonstrate that contiguous phage sequences had been captured with high fidelity, and little or no chromosomal contamination was evident in the PGSR phage collection.

### Comparative analysis of phage sequence recovery strategies

**Figure 3:**
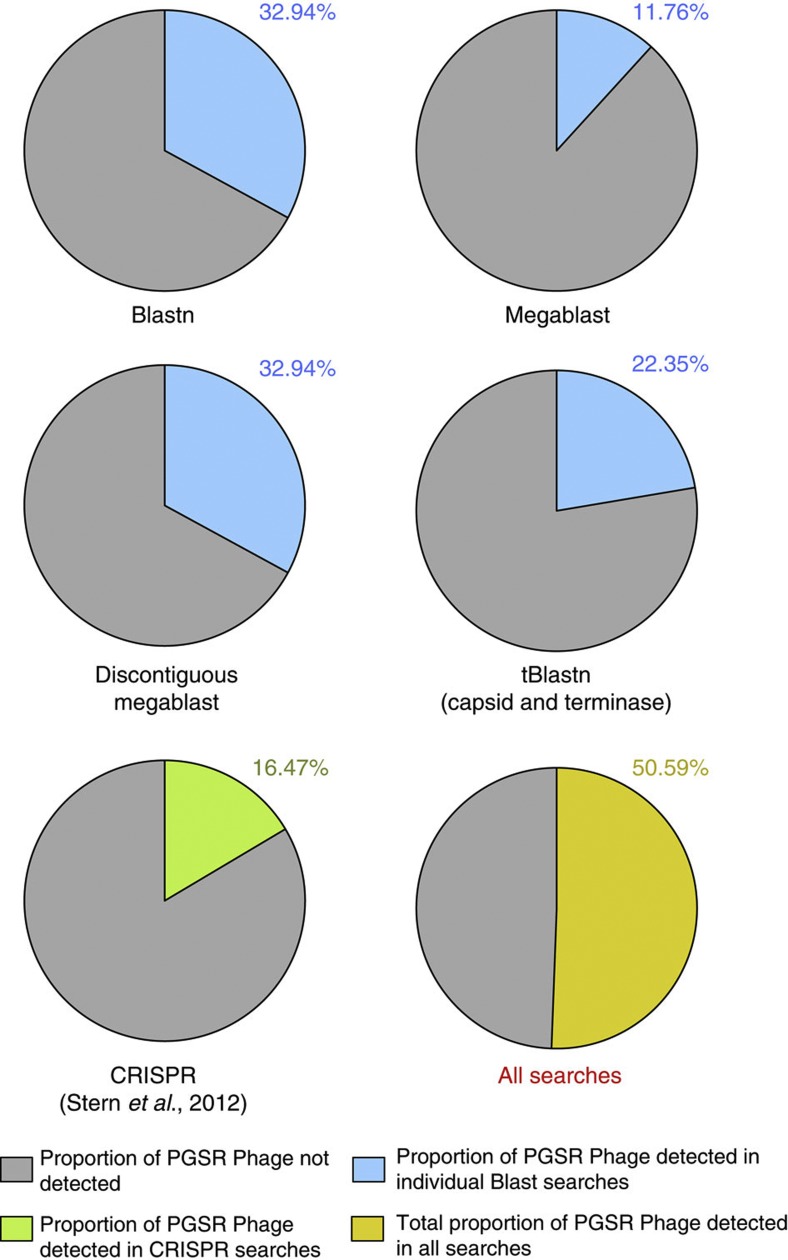
Recovery of PGSR phage sequences from metagenomic data sets. Commonly used alignment-driven approaches to analyse metagenomes were evaluated for their ability to identify PGSR phage sequences. The same metagenomic data sets surveyed using the PGSR approach were also subjected to a range of alignment-based searches, including gene-centric searches with unambiguous phage-encoded ORFs (capsid and terminase genes). In addition, 991 non-redundant phage contigs also identified in searches of these datasets by Stern *et al.,* using the recently developed CRISPR strategy, were compared^8^. Pie charts depicted show the proportion of PGSR phage sequences captured by each strategy, as well as the total proportion of PGSR phage identified by all strategies in combination (percentages shown). Blastn, Megablast, Discontiguous Megablast: show the proportions of PGSR phage captured in alignments with different blast algorithms when metagenomes were queried at the nucleotide level using whole-PGSR phage driver sequences (1e^−3^ or lower considered significant and retained). tBlastn: shows proportion of PGSR phage sequences identified using gene-centric surveys of metagenomes with all capsid and terminase genes encoded by driver sequences (1e^−3^ or lower considered significant). CRISPR: proportion of PGSR phage sequences identified in the 991 phage-like contigs identified by Stern *et al.*^8^, in recent surveys of the same metagenomes using CRISPR spacer regions. All searches: shows the total proportion of PGSR phage identified in the combined output of all searches conducted above.

In order to ascertain if the PGSR approach offers advantages over existing strategies for prophage-oriented analysis of metagenomic data sets, we assessed the ability of conventional alignment-driven approaches to also recover the PGSR phage sequences identified here. Although surveys of the same data sets using the same driver sequences with alignment-driven methods (Blastn and tBlastn) recovered a range of sequences not identified by the PGSR approach, alignment-based searches failed to detect the majority of phage sequences identified by the PGSR approach (Fig. 3).

In combination, all nucleotide-level searches with phage driver sequences identified 32.94% of PGSR phage sequences, with the majority of hits showing only low coverage of drivers, making a close relationship and a common host-range (that is, predicted bacterial host species) less likely to be a consistent feature of sequences recovered this way (Supplementary Table S2). Gene-centric surveys utilizing translated capsid and terminase ORFs from drivers identified only 22.35% of PGSR phage sequences (Fig. 3), but most hits exhibited relatively low levels of identity to driver sequence ORFs, again indicating the recovery of a more loosely related collection of contigs, with associated problems for host-range prediction (Supplementary Table S2).

Alternatively, Stern *et al.*^8^ have recently described an elegant strategy utilizing CRISPR spacer regions to identify phage sequences in metagenomic data sets, and also facilitate host-range prediction. This strategy has been applied to the same gut metagenomic data sets used here, but only 16.47% of the 85 PGSR phage were represented among the 991 phage sequences recovered using CRISPR spacers (Fig. 3). Collectively, these comparisons show the PGSR approach can identify phage or prophage sequences within metagenomes not readily detected by other approaches, and complement existing strategies to access viral metagenomes.

### Inference of host phylogeny

**Figure 4:**
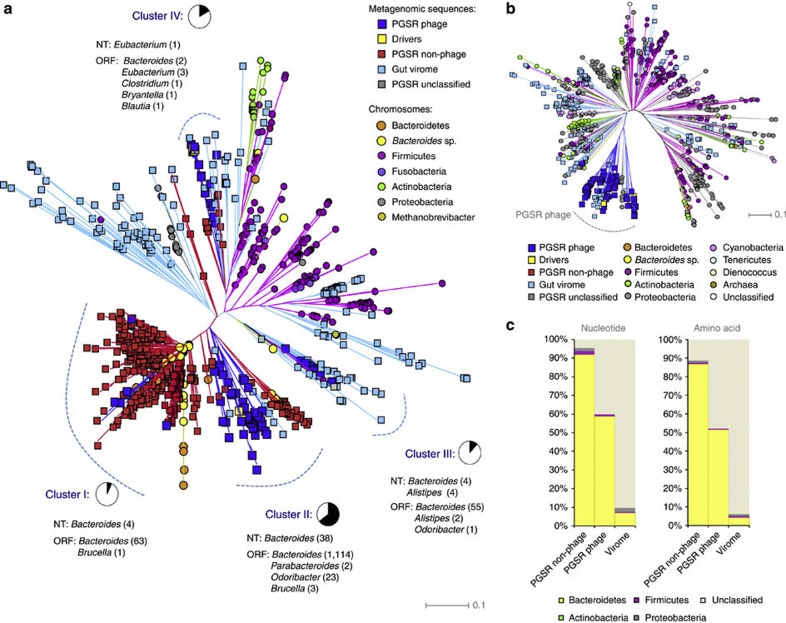
Inference of PGSR phage host-range. PGSR sequences were compared with a wide range of bacterial chromosomes and phage genomes, using both tetranucleotide profiles and alignment-based methods (Blast). (**a**) Phylogram showing relationships between PGSR sequences, human gut-associated chromosomes (*n*=324) and all large contigs from assembled gut viral metagenomes (*n*=188, 10 kb or over), based on tetranucleotide profiles. Clusters I–IV indicate regions populated by PGSR phage and driver sequences, and associated pie charts provide the proportion of total PGSR phage sequences in each cluster, designated by black segments. NT (nucleotide): shows genus-level taxonomic assignments for PGSR phage in each cluster based on Blastn searches, and figures in parentheses show total number of PGSR phage affiliated with each genus (≥75% identity, 1e^−5^ or lower, alignment length of 1 kb or more). ORF: shows genus-level taxonomic assignments for PGSR phage in each cluster based on tBlastn alignments of individual PGSR phage ORFs with 1,700 complete bacterial chromsomes (≥75% identity, 1e^−5^ or lower). Figures in parentheses show total number of PGSR phage ORFs affiliated with each genus listed. (**b**) Phylogram showing relationships between PGSR phage sequences, large fragments from gut viral metagenomes, and complete phage genomes (*n*=647 genomes, 10 kb or over), based on tetranucleotide profiles. For phage genome sequences assigned phylogeny reflects that of host species where known. Scale bars for parts **a** and **b** show distance in arbitrary units, and all phylograms represent the most probable topologies based on 200 bootstrap replicates. (**c**) Total proportion of PGSR sequences and viral metagenome contigs represented in part **a** affiliated to phylum-level taxonomic groups based on alignments against 1,821 bacterial and archaeal chromsomes. Nucleotide: shows the proportion of sequences affiliated to each phylum based on valid Blastn hits (minimum 75% identity over 1 kb or more, 1e^−5^ or lower). Amino acid: shows affiliation of all putative protein encoding genes from each data set based on tBlastn searches (minimum 75% identity or over, 1e^−5^ or lower). See also Supplementary Data 2. The source and further details of sequences used in the analyses presented in **a**–**c** is provided in Supplementary Table S1, Supplementary Data 3–6.

A major benefit of the PGSR approach should be an inherent inference of host-range for retrieved phage contigs, based on that of driver sequences. In order to confirm the integrity of this host-range affiliation, we explored the relationship of PGSR sequences with a broad cross section of chromosomal sequences and phage genomes. Initially, PGSR sequences were compared with a collection of 324 chromosomes from gut-associated bacteria, 647 complete phage genomes and 188 large contigs from gut virome assemblies, based on TUPs. Relationships were visualized by construction of phylograms, which showed a clear association of chromosomal sequences congruent with membership of major bacterial divisions in the gut microbiome (Bacteroidetes, Firmicutes, Actinobacteria and Proteobacteria) (Fig. 4a).

The majority of both PGSR phage and non-phage sequences were localized to four distinct regions of phylograms, designated Clusters I–IV (Fig. 4a). Most of these clusters were dominated by chromosomal sequences from gut-associated *Bacteroides spp*., and other closely related members of the *Bacteroidales,* with clusters I, II and III collectively accounting for 90.69% of all PGSR sequences, and 95% of all *Bacteroidales* chromosomes used (Fig. 4a). A distinct clustering of PGSR phage was also observed in phylograms constructed from TUPs of complete phage genomes and gut virome contigs (Fig. 4b), and with the exception of a single sequence, PGSR phage were most closely related to each other and confined to a distinct clade (Fig. 4b). The affiliation of PGSR sequences with the *Bacteroidales* was also retained when comparisons, were expanded to encompass a broader collection of bacterial chromosomes (*n*=1,700) from a wider range of habitats, and TUP-based affiliations examined using Emergent Self Organizing Maps (Supplementary Fig. S4).

To confirm the TUP-based phylogenetic inference for PGSR sequences, and the implied host-range for PGSR phage, alignment-based searches of 1,821 bacterial and archaeal chromosomes at both the nucleotide (Blastn) and ORF (tBlastn) level were also conducted. In both searches, PGSR phage sequences that could be classified based on homology to chromosome sequences (minimum 75% identity, 1e^−5^ or lower and over a minimum of 1 kb of query sequence for nucleotide alignments) were almost exclusively associated with members of the genus *Bacteroides* and mapped to all regions of phylograms populated by PGSR phage (Fig. 4a, Supplementary Data 2). Furthermore, TUP-based host-range predictions were also supported by phylogenetic affiliations of contigs undertaken by Stern *et al.*^8^, in CRISPR-based surveys of the MetaHIT data set^21^. In cases where PGSR phage contigs were identified and affiliated independently by Stern *et al.*^8^, host-range associations were comparable, and in most cases identical to, those assigned in the present study (Supplementary Data 2).

Of the classifiable PGSR phage sequences not affiliated with *Bacteroides* *spp.* by alignments (nt alignment; *n*=5, 10%), the majority were associated with the genus *Alistipes* (*n*=4), also a member of the gut-associated *Bacteroidales*, and terminase genes from *Bacteroidales* phage drivers have also previously been shown to be closely related to those associated with *Alistipes sp*.^13^ (Supplementary Fig. S1). Conversely, only a small number of PGSR phage sequences (*n*=3; 3.5%), and several PGSR non-phage sequences (*n*=11; 3.43%) were affiliated with non-*Bacteroidales* species in alignments (Fig. 4c, Supplementary Data 2). Overall, these analyses indicate that the PGSR approach is able to acquire phylogenetically targeted and closely related phage sequences from metagenomic data sets, and provide a strong indication of host-range taxonomy.

### Habitat affiliation of *Bacteroidales*-like PGSR phage

**Figure 5:**
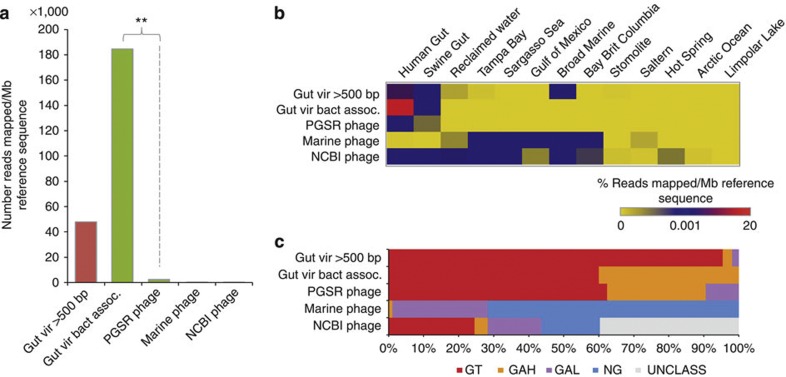
PGSR phage representation in human gut viral metagenomes. The representation of PGSR phage sequences in existing gut viral metagenomes, as well as viral and chromosomal metagenomes from other habitats, was assessed and compared with other phage sequence sets. (**a**) Representation of phage sequence sets in human gut viral metagenomes^11^. Individual pyrosequencing reads were mapped to respective phage sequence sets with high stringency (a minimum of 90% identity over 90% of the read). The number of reads mapped was normalized for size of reference data sets (expressed as reads mapped/Mb reference sequence). (**b**) Heat map showing relative representation of PGSR phage and other phage sequence sets in viromes from gut and non-gut habitats. Reads from each virome were mapped to reference phage sequence sets as for part **a**, but using low stringency criteria (minimum 70% identity over 25% of the read). The percentage of reads mapped was normalized for size of reference data sets (expressed as % reads mapped/Mb reference sequence). (**c**) Proportion of phage with homology to sequences in standard metagenomes and virome assemblies, derived from gut and non-gut habitats. Phage sequences from each collection were used to search metagenomic data sets with Blastn, and valid hits (minimum 75% identity over 100 nt or more, 1e^−5^ or lower) were used to assign each sequence to one of five categories. GT (gut): phage sequences producing valid hits only in gut data sets; NG (non-gut): phage sequences producing valid hits only in non-gut data sets; GAH (gut-associated high): phage sequences producing valid hits in both gut and non-gut data sets, but with the majority derived from gut metagenomes. GAL (gut-associated low): phage sequences generating valid hits in both gut and non-gut data sets, but with the majority originating from non-gut metagenomes; UNCLASS: sequences producing no valid hits in any metagenome examined. Gut vir >500 bp—all contigs from human gut virome assemblies over 500 bp in length; Gut vir bact assoc.—all contigs from human gut virome assemblies affiliated with *Bacteroidales* driver sequences based on PGSR search criteria (as used to identify PGSR phage sequences in gut metagenomes); PGSR phage—all 85 *Bacteroidales*-like PGSR sequences classified as phage; marine phage—99 phage genome sequences from marine phage; NCBI phage—612 complete phage genomes available from the NCBI phage refseq collection. **P≤0.01 (*χ*^2^-test). Details of viromes, metagenomes and phage genomes utilized are provided in Supplementary Table S1, Supplementary Data 3–6.

In order to determine whether the *Bacteroidales*-like PGSR phage captured here are already well represented in existing gut viral metagenomes^11^, pyrosequencing reads from gut viromes were mapped to the PGSR phage sequence set with high stringency (minimum 90% identity over 90% of sequence read). The proportion of reads recruited was then used to estimate levels of PGSR phage representation in viral data sets. Sequences mapping to PGSR phage contigs were found to be poorly represented in these data sets, when compared with *Bacteroidales*-like phage contigs assembled from the same gut virome reads (also identified by applying the PGSR approach to virome assemblies) (Fig. 5a). Given that the original analysis of these viromes also indicated phage associated with the *Bacteroidales* to be well represented^11^, this supports a specific under-representation of PGSR phage homologues in these data sets, rather than a paucity of *Bacteroidales*-like phage in general.

To explore the distribution of PGSR phage in other habitats, we next investigated their representation in a range of additional viromes and metagenomes (Fig. 5b,c). Using 13 viral metagenomes derived from gut and non-gut environments (Supplementary Table S1), we again mapped pyrosequencing reads to PGSR sequences, this time using a low stringency set of criteria (minimum 75% identity over 25% of sequence read) to provide the most conservative estimates of phage distribution. To further expand the range of habitats and ecosystems evaluated, the presence of sequences homologous to PGSR phage was also assessed in 12 conventional metagenomes and 2 virome assemblies (Fig. 5b,c; Supplementary Table S1). For these assembled data sets, the results of Blast searches were used to classify each phage sequence based on the hit rate in gut and non-gut metagenomes (also using relaxed search criteria to afford conservative estimates of phage habitat affiliation). These surveys indicated a clear association of PGSR phage and virome contigs with the human gut microbiome, and a comparative rarity of homologous sequences in non-gut data sets (Fig. 5b,c).

### Functions and lifestyle of *Bacteroidales*-like PGSR phage

**Figure 6:**
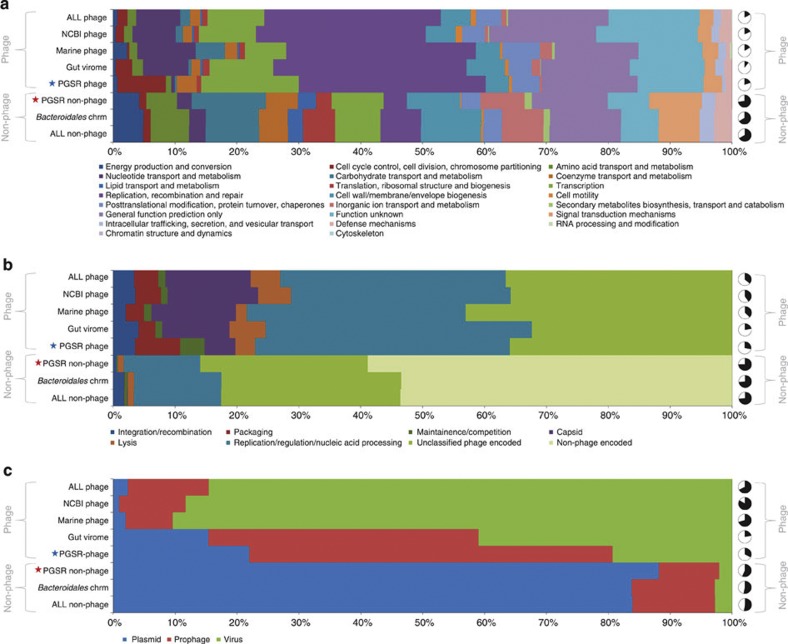
Functional profiles of PGSR sequences. The functional profiles of PGSR phage and non-phage sequences were compared with those found in phage genomes (*n*=711), gut virome fragments (all contigs assembled from 12 individual gut viromes^11^), and 70 chromosomes from gut-associated *Bacteroidales* species (See Supplementary Table S1, Supplementary Data 3–6 for source and details of sequence data). Amino-acid sequences from all predicted ORFs in each data set were used to search the COG^26^ database, the CDD^25^, and the ACLAME database^27^. The proportion of assignable ORFs affiliated to distinct categories in each database is displayed in horizontal bars, and associated pie charts show the total proportion of ORFs in each sequence set generating valid hits in database searches (black segments). (**a**) Results from searches of the COG database, showing proportions of ORFs assignable to COG classes. (**b**) Results for searches of the CDD, showing proportions of ORFs encoding conserved domain architectures related to phage and non-phage associated functions. (**c**) Results from searches of the ACLAME database, showing proportions of ORFs generating valid hits to genes encoded by distinct types of mobile genetic element represented in the database (plasmid, virus and prophage). All phage shows combined results from PGSR-phage, NCBI phage, Marine phage and Gut virome fragments. All non-phage shows combined results from PGSR non-phage and *Bacteroidales* chromosomes. Stars highlight the position of PGSR phage and non-phage sequences in charts.

To examine the activities encoded by these novel *Bacteroidales*-like PGSR phage sequences, and compare their functional profiles with other phage and chromosomal sequence collections, we next used predicted ORFs from all PGSR contigs to search the Conserved Domain Database (CDD)^25^, the Clusters of Orthologous Groups database (COG)^26^, and the A CLAssification of Mobile Genetic Elements database (ACLAME) of MGE-encoded genes^27^ (Fig. 6). Collectively, these search results further supported the provenance and classification of PGSR sequences as phage or non-phage, and the fidelity of the PGSR approach for recovery of phage genome fragments from conventional metagenomes (Fig. 6).

COG and CDD functional profiles showed striking differences between PGSR phage and non-phage, with PGSR phage profiles congruent with a viral lifestyle and enriched in genes involved in capsid structure, host lysis, genome packaging, transcription, as well as replication and recombination (*P*≤0.004, *χ*^2^-test; Fig. 6a,b). As expected for viral genomes, COG profiles from PGSR phage sequences also showed a general lack of functions associated with energy production, nutrient metabolism and transport (amino acids, lipids and carbohydrates), cell wall and membrane biogenesis, and ribosome production and translation (*P*≤0.01, *χ*^2^-test; Fig. 6a).

Although some differences were observed between individual phage sequence sets (Marine phage, NCBI phage and gut virome contigs), overall, the functional profile of PGSR phage was comparable to the other phage sequence collections analysed, while the PGSR non-phage functional profile was similar to that obtained from *Bacteroidales* chromosomes (Fig. 6a,b). However, despite the similarities in functional profiles between phage sequence sets, surveys of the ACLAME database of MGE-encoded genes indicated marked differences in the prevailing lifestyle of human gut-associated phage, as compared with other phage sequence collections (Fig. 6c). Assignable sequences in the ACLAME database from PGSR-phage and gut virome contigs were predominantly associated with prophage, in stark contrast to other phage sequence collections (*P*≤0.001, *χ*^2^-test; Fig. 6c). In keeping with these observations, 23.5% of PGSR phage contigs were identified as encoding integrases or site-specific recombinases based on CDD searches. The dominant conserved domain model among these proteins was the DNA_BRE_C superfamily (cd00379), which includes phage Lambda integrase and phage P1 Cre recombinase.

**Figure 7:**
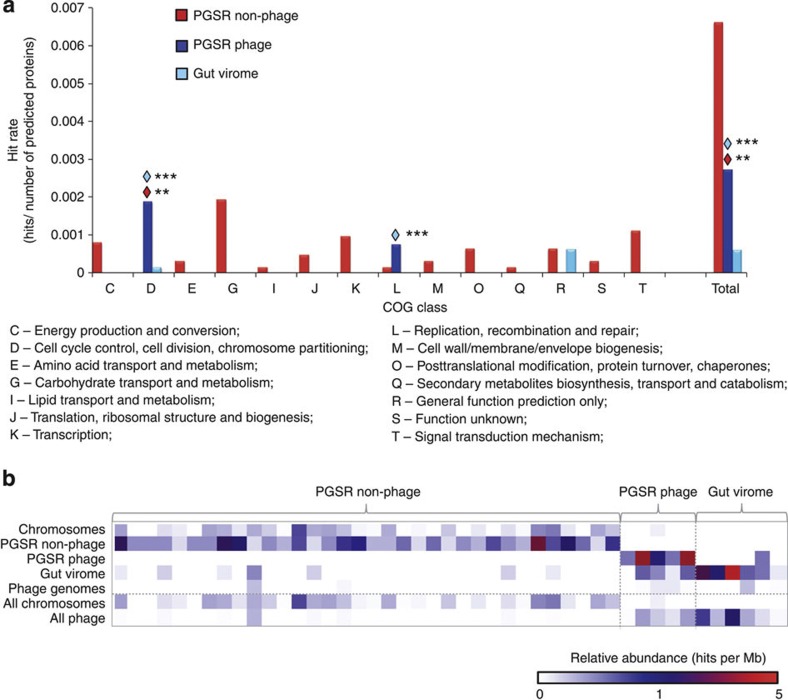
Representation of PGSR phage sequences in the human gut metaproteome. To further explore the functional profile of PGSR *Bacteroidales*-like phage, and their contribution to the human gut metaproteome, a shotgun metaproteome was generated from a human faecal microbiome and the resulting 177,729 mass spectra used to search custom databases of all putative proteins encoded PGSR phage, PGSR non-phage and VLP-derived contigs from human gut viral metagenomes^11^. (**a**) Shows relative hit rates in the gut metaproteome, for amino-acid sequences originating in each data set used to query mass spectra (PGSR phage, PGSR non-phage, VLP-derived gut virome). Relative hit rates were calculated by normalizing the number of proteins from each data set detected in the gut metaproteome by the total number of ORFs in parental data sets (expressed as hits per total number of predicted proteins in each data set). Symbols above bars indicate statistically significant differences in relative hit rate with the data set of corresponding symbol colour (**P=0.01 or lower; ***P=0.001 or lower; *χ*^2^-test). Putative functions of identified proteins were based on COG searches (1e^−2^ or lower; Supplementary Table S3). (**b**) Heat map shows relative abundance of sequences homologous to those detected in the gut metaproteome, within a broad cross section of bacterial and archaeal chromosomal sequences (*n*=1,821, PGSR non-phage), and phage sequences (711 phage genomes, PGSR phage sequences and assemblies of human gut viromes), expressed as hits per Mb DNA^48^,^49^ (valid hits=minimum 35% identity over 30 aa or more, 1e^−5^ or lower). See Supplementary Table S1, Supplementary Data 3–6 for sources and details of sequences used.

To further explore the functional profile of PGSR *Bacteroidales*-like phage, we used mass spectrometry to generate a shotgun metaproteome from a human faecal microbiome, and used the derived 177,729 mass spectra to search custom databases of all putative proteins encoded by PGSR *Bacteroidales-*like sequences (phage and non-phage), and all contigs from VLP-derived human gut viral metagenome assemblies^11^. Proteins from all data sets were identified in the metaproteome, but as expected, proteins derived from PGSR non-phage sequences (presumed to be chromosomal in origin) constituted the majority of matches (Fig. 7a, Supplementary Table S3).

Phage-associated proteins detected represented just three COG classes (cell cycle control; replication, recombination and repair; general function prediction) (Fig. 7a). This is in contrast to 13 COG classes represented by metaproteome hits from non-phage PGSR fragments, which included many proteins with activities linked to carbohydrate metabolism, a major activity of gut microbes and in particular *Bacteroides* *spp.*^21^,^28^,^29^ (Fig. 7a). When relative abundance of homologous ORFs was assessed in a broader range of phage genomes and chromosomes, a distinct functional separation was also apparent between phage and non-phage sequences (Fig. 7b). Phage-associated metaproteome hits showed a high relative abundance in phage genomes and other phage sequences, but were poorly represented in chromosomal sequences, with the converse true for PGSR non-phage proteins (Fig. 7b).

The predicted activities of viral-encoded proteins detected in the metaproteome were also congruent with a lysogenic viral lifestyle, and associated with stability and maintenance of phage genomes in host bacteria (DNA methylases, partitioning proteins, site-specific recombinases/integrases; Supplementary Table S3). DNA methylases are frequently deployed by phage for protection from host defence systems by preventing degradation from host endonucleases through DNA methylation, and may also be involved in stable lysogeny^30^,^31^. Site-specific recombinases/integrases and partitioning systems are also features of temperate phage and associated with the lysogenic cycle^11^,^32^. Overall, the results of these surveys fit well with recent studies of the gut virome indicating a dominance of temperate phage^7^,^11^, and show that predominantly lysogenic phage (most likely in the form of prophage) have been accessed by the PGSR approach.

### *Bacteroidales*-like PGSR phage encode functional β-lactamases

Functional profiling of PGSR phage sequences also indicated that these encode activities of direct relevance to human health, in the form of antibiotic resistance genes. In total, 12 PGSR phage sequences were found collectively to encode five putative β-lactamase variants exhibiting high levels of identity to each other (designated type 1–5; Supplementary Table S4). These sequences were most closely related to predicted metallo-β-lactamases from *Bacteroides sp.* D22, *Bacteroides sp.* 1_1_30 and *Bacteroides stercoris,* but showed no significant homology to entries in the Antibiotic Resistance Genes Database^33^ (minimum 20% identity, 1e^−2^ or lower).

To confirm the functionality of these putative resistance determinants, corresponding regions of PGSR phage were amplified from total gut metagenomic DNA, cloned and expressed in *E. coli.* Transformants were then tested for their susceptibility to a range of β-lactam antibiotics. Only Type-2 PGSR phage-encoded β-lactamases were successfully amplified and cloned, but were capable of conferring resistance against mecillinam (Supplementary Fig. S5), a member of the amidinopenicillin family with high affinity for Gram-negative penicillin-binding protein 2, but little activity against Gram-positive bacteria^34^. This antibiotic is not widely used in many European countries or the USA, but has been identified as potentially useful in the treatment of multi-drug resistant infections caused by Gram-negative species^35^. As such, identification of viable mecillinam resistance genes circulating among lysogenic *Bacteroides* phage in the gut mobile metagenome is of particular significance, and highlights the potential for dissemination and spread of these resistance determinants via horizontal gene transfer.

### Inter-individual variation in *Bacteroidales*-like phage carriage

**Figure 8:**
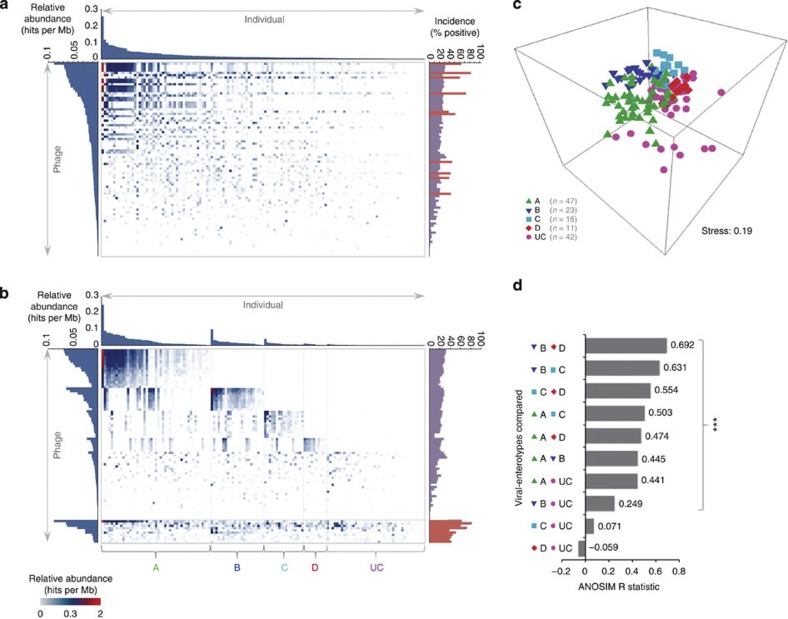
Inter-individual variation of *Bacteroidales*-like viral-enterotypes. Inter-individual variation in carriage of PGSR phage and related sequences was assessed by calculating relative abundance of sequences with homology to PGSR phage in individual gut metagenomes (minimum 80% identity over 50% of subject sequence, 1e^−5^ or lower). (**a**,**b**) Heat maps illustrating relative abundance of PGSR phage sequences in human gut metagenomes. Columns represent individual metagenomes and rows represent PGSR phage sequences. Intensity of shading in each cell indicates relative abundance of sequences homologous to each PGSR phage sequence, in each individual metagenome (hits per Mb). Associated histograms show average relative abundance of homologues to each PGSR phage sequence across all individuals (left histogram), average relative abundance of all PGSR phage homologues per individual (top histogram), and incidence of sequences homologous to each PGSR phage sequence as a % of positive metagenomes (Right histogram). Map **a** shows results ranked by average relative abundance across all PGSR phage and individuals. Map **b** shows results of heuristic hierarchical grouping of individuals based on phage relative abundance profiles into ‘viral-enterotypes’ A, B, C, D or unclassified (UC). The most broadly distributed PGSR phage (with an incidence of 40% or over), shown in the lower segment of this heat map, were not utilized for heuristic ranking. (**c**) The validity of putative viral-enterotypes was tested by ordination of individual relative abundance profiles using unsupervised non-metric MDS. Points represent individual gut metagenomes, and colours correspond to viral-enterotypes assigned in heat map **b**. (**d**) Shows values for the ANOSIM *R* statistic obtained from comparisons of groupings obtained in MDS plots (part **c**), which indicates increasing separation of groups as values approach 1. *** Denotes significant separation between groups (*P*=0.002). The sources of human gut metagenomes used in these analyses are provided in Supplementary Table S1.

To assess inter-individual variation in carriage of PGSR phage and related sequences, we calculated the relative abundance of sequences homologous to PGSR phage in individual gut metagenomes (minimum 80% identity over 50% of the subject sequence, 1e^−5^ or lower). This indicated that such sequences are broadly distributed among the gut microbiomes examined (Fig. 8a), with the incidence of PGSR homologues ranging from 51.8–82.73% of metagenomes for the five most broadly represented PGSR phage (encompassing both Japanese and European individuals) (Fig. 8a). Notably, these apparently broadly distributed virotypes included sequences with homology to PGSR phage harbouring type-2 β-lactamases with proven function.

Heat maps of relative abundance data also suggested the existence of several distinct patterns of *Bacteroidales*-like phage carriage shared by multiple individuals (Fig. 8a). To investigate this further, we employed a heuristic hierarchical ranking approach, to progressively group individual microbiomes based on phage relative abundance profiles. This simple strategy revealed four distinct variants of *Bacteroidales*-like phage relative abundance profiles across individual metagenomes, designated ‘viral-enterotypes’ A–D (Fig. 8b). The validity of these putative phage-oriented microbiome groupings was subsequently confirmed using unsupervised ordination by non-metric multi-dimensional scaling (MDS) and analysis of similarities (ANOSIM) (*P*=0.002; Fig. 8c,d). However, much overlap was evident between individual groups in all analyses, and not all groups were significantly or clearly separated (Fig. 8c,d). These observations are reminiscent of the enterotypes model recently reported by Arumugam *et al.*^36^ in which members of the *Bacteroidales* also featured as drivers of the observed enterotypes^36^.

## Discussion

### 

Bacteriophage genomes are believed to coevolve with, or adapt to long-term bacterial hosts, leading to the development of nucleotide usage patterns that resemble those of the host chromosome^22^,^23^,^24^,^37^. Here we show that global TUPs, in conjunction with functional profiling, can be employed for the direct phage-oriented dissection of conventional metagenomes, permitting the resolution and host-range affiliation of subliminal virome fractions contained within. A major advantage of the use of genome signatures in this application is the gene-independent, alignment-free nature of this approach. As nucleotide signatures are generally pervasive across genomes^23^,^37^, the requirement for the presence of conserved genes or motifs typically used for identification and classification of sequences is circumvented.

As such, genome signatures are well suited to analysis of sequence types lacking robust and universally conserved phylogenetic anchors, and fragmentary data sets where conventional gene-centric alignment-driven methods often perform poorly^37^,^38^,^39^,^40^,^41^,^42^. Metagenomes, and phage (or other MGE sequences) captured within, constitute prime examples of such data sets and sequence types, with the PGSR approach shown to resolve phage sequences not readily detected by conventional alignment-driven approaches, even when used in conjunction with phage-related sequence motifs or genes.

However, this method does not overcome all disadvantages of metagenomic approaches for viral discovery. For example, the focus on acquisition and analysis of chromosomal DNA in conventional metagenomic data sets will exclude RNA phage, and there remains a need for continued culture-based isolation of phage to provide well-characterized driver sequences. Despite these caveats, the PGSR approach can recover many additional phage sequences from few initial driver sequences, access phage not well represented in VLP-based censuses, and potentially be used to mine metagenomes for other MGE and semi-conserved sequences.

Furthermore, the use of well characterised phage sequences with known host-ranges, as drivers in the PGSR approach, permits recovery of contigs with a common taxonomic imprint, automatically providing an indication of host phylogeny. A high level of congruence between TUP inferred phage–host associations, and established host ranges for cultivable bacteria and their phage has previously been demonstrated^23^, and also indicated to hold true for viral sequences represented in metagenomic data sets^37^. Importantly, previous genome signature-based analyses of whole-community shotgun metagenomes have shown that the shared selective pressures placed upon microbes occupying a given habitat do not obscure the taxonomic imprint rooted in TUPs, even when the community is subject to strong and constant environmental stress, the genus-level resolution of metagenomic fragments remains feasible^37^. These observations are exemplified by the clear and consistent association of PGSR acquired contigs with *Bacteroides* spp. and members of the wider *Bacteroidales* in the present study.

Conversely, a small number of PGSR phage sequences (*n*=3) were affiliated with non-*Bacteroidales* species in alignment-driven surveys, and mapped to regions of phylograms closely related to members of the *Clostridiales,* but also populated by a mixture of *Bacteroidales*-affiliated and unaffiliated sequences. This variegated phylogenetic signal could be the result of convergent evolutionary processes that generate similar TUPs in unrelated organisms or phage genomes, obscuring the taxonomic imprint and leading to spurious host-range affiliations^22^,^23^. There is also the possibility that these sequences represent examples of viruses with very broad host-ranges^43^, or those in the process of adapting to new host species. Alternatively, the acquisition of new genetic material by horizontal gene transfer in phage is also well documented, and could account for the discordant alignment-based affiliations of the PGSR sequences in question. These issues are not unique to genome signature-based approaches and are also important considerations in gene-centric taxonomy^22^,^23^, constituting a potential limitation in both strategies.

The utilization of standard metagenomes in the PGSR approach should also provide access to fractions of bacteriophage communities that may be poorly represented by other methods. In light of the reported dominance of temperate phage in the human gut ecosystem^7^,^11^, it would be expected that greater access to quiescent phage will be important in further exploration of this viral community and will yield much insight into its structure and function. As such it is notable that the PGSR phage captured here were indicated to be predominantly prophage, and not well represented in existing VLP-derived gut viral data sets, supporting the identification and analysis of phage sequences not readily accessed by other approaches. However, variation in the geographic origins of the metagenomes and viromes utilized for these analyses cannot be excluded as a possible factor in the low level of PGSR phage representation in VLP-based data sets, with gut metagenomes from which PGSR phage were retrieved European in origin, but viral data sets generated from American individuals^11^,^13^,^21^. Alternatively, phage sequences recovered here may mostly represent inactivated prophage, which no longer contribute to the active, extrinsic VLP pool sampled in other studies.

Subsequent analyses showed PGSR phage not only encode functions directly relevant to human health (reinforcing the role of phage in spread of antibiotic resistance determinants) but also the potential specificity of PGSR phage to the human gut habitat, which is relevant to biotechnological applications of phage such as microbial source tracking^13^,^44^. In addition, the possible existence of ‘viral-enterotypes’ in this region of the gut virome was also revealed when individual gut metagenomes were compared. The phage-oriented grouping of microbiomes is reminiscent of the enterotypes model recently reported by Arumugam *et al.*^36^, where individuals were grouped based on similarities in microbiome composition. Notably, two of the three microbial enterotypes presented by Arumugam *et al.*^36^ were driven by members of the *Bacteroidales* (*Bacteroides* and *Prevotella*), and it seems logical that examination of gut-specific temperate phage associated with these genera should generate concordant findings.

However, the *Bacteroidales*-like phage-oriented microbiome groupings observed here appear less well-defined and may be indicative of inter-individual gradients in phage population structure rather than entirely discrete groupings (as has also been posited for microbial enterotypes). Moreover, the grouping of individuals based on virome structure is inconsistent with other recent studies of the gut virome, where no such associations were observed^7^,^8^,^11^. These discrepancies may be due to the phylogenetically targeted analysis afforded by the PGSR approach coupled with the nature of the data sets from which PGSR phage are derived. In conjunction, these attributes should provide access to a closely related population of predominantly lysogenic phage (as prophage), expected to represent a more stable region of the phage ecological landscape in the gut microbiome.

Collectively, these factors could permit resolution of inter-individual similarities in gut virome structure obscured in studies focused on the virome as a whole, or the free, replicating virome fraction accessed through VLP libraries. Nevertheless, the data sets utilized here present only a ‘snapshot’ of the gut microbiome and do not capture the temporal dynamics of phage–host interactions. Much scope also remains to refine criteria and strategies used to identify and explore these putative viral-enterotypes. Although our observations provide the first indication that such groupings may exist in the gut virome, it is clear that further work will be required to confirm or refute the potential existence of viral-enterotypes within the *Bacteroidales* phage gene-space, and their significance, if any, for ecosystem function and development.

Overall, in this study we have validated a new strategy for analysing and understanding the composition of metagenomic data sets, as well as exploring and interpreting microbial viromes. This simple and accessible approach augments existing strategies, and can be applied retrospectively to available metagenomes to rapidly expand our knowledge of phage communities. Here we have employed the PGSR method to dissect human metagenomes with phylogenetic precision, and provide further insight into the structure and function of the human gut virome.

## Methods

### Phage genome signature-based dissection of gut metagenomes

To identify potential *Bacteroidales*-like phage sequences in human gut metagenomes, contigs from each data set were subject to genome signature comparisons with driver phage sequences, and subsequent binning based on encoded functions as outlined in Fig. 1. Correlations between global usage patterns of all 256 possible tetranucleotide sequences in driver phage sequences (Table 1, Supplementary Fig. S1), and all large contigs from human gut metagenomes^21^,^28^,^45^ (Supplementary Table S1), were calculated according to the method of Teeling *et al.*^46^, using the standalone TETRA 1.0 program. To ensure unambiguous tetranucleotide profiles were generated and recovered phage sequences could be distinguished, all metagenome contigs utilized were 10 kb or over in length^7^,^46^. All sequences were extended by their reverse complement, and the divergence between observed and expected frequencies for each tetranucleotide were converted to *Z*-scores, which were compared pairwise between sequences to generate a Pearson’s similarity matrix of tetranucleotide usage correlation scores^46^. Metagenomic sequences exhibiting tetranucleotide correlation values of 0.6 or over^13^ to any phage driver sequence were retained and protein encoding genes predicted using the RAST server, accessed through the myRAST interface^47^. For each metagenomic sequence, functional profiles were subsequently obtained by searches against the CDD^25^ (1e^−2^ or lower), using amino-acid sequences from predicted ORFs, and used to categorize each retrieved metagenomic contig as phage, non-phage or unclassified (UC) based on the following criteria: (i) phage: contains at least one unambiguous phage-related gene (for example, capsid, terminase, tail fibre, or annotated as phage related) and/or at least one phage-related ORF also present in one or more driver sequences; (ii) non-phage: absence of phage-related ORFs and/or dominated by ORFs-encoding functions commonly associated with chromosomal sequences; and (iii) UC: no ORFs with functions that provide clear indication of putative sequence type.

### Annotation of PGSR phage sequences and designation of ORFs

Randomly selected PGSR phage sequences (*n*=20; Fig. 2a) were annotated in Geneious 5.6.5 based on ORF predictions as described above. Amino-acid sequences for each ORF were used to search custom databases representing a broad collection of phage sequences using tBlastn (711 phage genomes and all contigs assembled from human gut viral metagenomes^11^), as well as the CDD^25^. Valid hits to other phage sequences (1e^−3^ or lower), or the presence of conserved domains (1e^−2^ or lower) with phage-related functions, were used to identify phage-related ORFs in each sequence (Fig. 2a).

### Calculation of ORF relative abundance

The relative abundance of ORFs in an extensive collection of chromosomal sequences (1,821 bacterial and archaeal chromosomes and all PGSR non-phage) as well as all phage sequences (711 phage genomes, viral metagenome assemblies and PGSR phage), was carried out as described previously^48^,^49^. Briefly, translated amino-acid sequences for each ORF were used to search data sets using tBlastn, and valid hits (minimum 35% identity over 30 aa or more, 1e^−5^ or lower) used to calculate the relative abundance of each ORF in different data sets, expressed as hits per Mb (Fig. 2b). Significant differences between relative abundances were assessed using the *χ*^2^-test. Data sets and sequences utilized are described in Supplementary Table S1, Supplementary Data 3–6.

### Alignment-driven survey of PGSR phage–host phylogeny

To compare the PGSR approach with conventional alignment-driven methods, for recovery of sequences closely related to driver phage, all large metagenome contigs (10 kb and over) were also searched using a variety of blast algorithms (Blastn, megablast, discontiguous megablast, tBlastn), with phage driver sequences as queries for nucleotide-level searches, and driver encoded capsid and terminase amino-acid sequences as queries for ORF level searches (Supplementary Fig. S1, Supplementary Table S1). Blast searches were run with default parameters in all cases and implemented in Geneious 5.6.5 (Biomatters Ltd). All hits generating *e*-values of 1e^−3^ or lower in each search were considered valid and the resulting search results were made non-redundant, with only the best hit (based on bit score) for each subject sequence retained. The resulting data were then used to calculate the number of sequences recovered, average % identity, and average % query coverage, as well as to identify the proportion of PGSR phage sequences identified in each blast search.

### Clustering of sequences based on tetranucleotide usage

To test the phylogenetic inference afforded by the PGSR approach, PGSR sequences were compared with a selection of gut-associated chromosomal sequences (*n*=324) representing all major phylogenetic groups in the gut microbiome, and a large collection of phage genome sequences (*n*=647), as well as all large contigs from an independent assembly of 12 human gut viromes originally generated by Reyes *et al.*^11^ (*n*=188; Supplementary Table S1, Supplementary Data 3–6). All sequences utilized in this analysis were 10 kb in length of over. TUPs were calculated from all sequences as described above, using TETRA 1.0 (ref. 46). For calculation of TUPs from draft chromosomes, contigs were first concatenated before analysis using TETRA^13^. Pearson’s dissimilarity matrices generated from TUPs were subsequently used to construct phylograms with the neighbor-joining algorithm in PHYLIP 3.69 (ref. 50). Bootstrap analysis was performed based on methods described previously^22^, and conducted by sampling with replacement for each of the 256 TUPs, to produce 200 bootstrap replicates that were used to resolve the most probable topologies for each phylogram in Geneious 5.6.5. The final phylograms were visualized and annotated using Dendroscope 3.0.1 (ref. 51).

### Alignment-based affiliation of PGSR sequences

Alignments of PGSR phage nucleotide sequences and translated ORF sequences were conducted using Blastn and tBlastn, respectively, implemented in Geneious 5.6.5 and run with default parameters. PGSR sequences were compared with custom blast databases of 1,821 bacterial and archaeal chromosomal sequences from the NCBI and Human Microbiome Project (see Supplementary Table S1, Supplementary Data 3,4 for details and source of sequences). Only hits with 75% identity or over, and *e*-values of 1e^−5^ or lower were considered valid. For nucleotide-level searches, alignments were also required to cover a minimum of 1 kb of PGSR query sequence to be considered valid. Top hits for each query (by bit score) were then used to affiliate each PGSR phage sequence or ORF with a bacterial genus (Supplementary Data 2) or order (Fig. 4c). For taxonomic affiliation, ORF homologies were utilized only where no valid nucleotide-level alignments were generated (Supplementary Data 2). Where only ORF-based affiliation was considered, a minimum of two ORFs within a PGSR phage sequence were required to produce valid hits to bacterial species derived from the same order (Fig. 4c, Supplementary Data 2). PGSR phage sequences were also compared with all phage-like sequences from the MetaHIT^21^ data set independently identified by Stern *et al.*^8^, and the host ranges they inferred for those sequences based on Blastn alignments or CRISPR spacer analysis (Supplementary Table S2, Supplementary Data 2).

### Representation of PGSR phage sequences in human gut viromes

To assess the level of representation of PGSR phage sequences in existing human gut viral metagenomes, pooled pyrosequencing reads from 12 human gut viromes^11^ were mapped against PGSR phage sequences. Pyrosequencing reads were obtained from the NCBI short read archive and processed using CAMERA^52^ workflows as previously described by Ogilvie *et al.*^13^ Briefly, low-quality reads and duplicates were removed using the 454 QC and 454 duplicate clustering workflows, respectively, with default parameters. The resulting collection of high-quality reads were mapped against PGSR phage sequences, and other phage sequence collections using the Geneious 5.6.5 map to reference tool with the following criteria: a minimum of 90% identity over 90% of the read length, and a maximum of 10% mismatches per read with no gaps permitted. Each read was only permitted to map to a single reference sequence per data set. For each reference data set, the total number of reads mapped to all sequences with the reference set was then normalized by the total size of the reference sequence data set in question, to provide reads mapped/Mb reference data. Significant differences in the proportion of reads mapping to distinct reference sequence sets were identified using the *χ*^2^-test.

### Habitat affiliation of PGSR phage sequences

To investigate the representation of PGSR phage sequences in other habitats, both viral metagenomes and conventional metagenomic data sets were surveyed (Supplementary Table S1). For viral metagenomes, individual pyrosequencing reads were again mapped against PGSR phage and other reference data sets as describe above, but using relaxed criteria to afford conservative estimates of phage distribution: 70% identity over 25% of the read length, with a maximum of 10% mismatches and 10% gaps permitted per read. The percentage of reads from each virome mapping to a reference data set were normalized by reference data set size, as described above. In addition, assemblies of 12 conventional metagenomic data sets representing non-gut (terrestrial, freshwater and marine) and gut habitats, as well as 2 assembled viral metagenomes (Supplementary Table S1), were also analysed for sequences with homology to PGSR and other phage. In this latter analysis, phage sequences were used to search each data set using Blast, and the number of valid hits from gut and non-gut metagenomes (minimum of 75% identity over 100 nt or more, *e*-value or 1e^−5^ or lower) calculated, normalized by collective size of associated metagenomes, and used to affiliate each phage sequence to one of four categories based on relative representation in gut and non-gut data sets.

### Functional profiling

For analysis of functions encoded by PGSR phage and non-phage sequences, all protein encoding genes in both sequence sets were annotated using the RAST server as described above, and amino-acid sequences from each group of sequences used to search the CDD^25^, the COG^26^, and the ACLAME databases^27^. Hits generating *e*-values of 1e^−2^ or lower were considered valid in searches of CDD and ACLAME databases, and 1e^−3^ or lower in COG searches. Valid hits were then used to compare functional profiles of PGSR sequences with other sequence sets. Comparisons were made at the Class level for COG searches, and element type (plasmid, virus and prophage) for ACLAME searches. For CDD searches, conserved domains detected in phage ORFs were binned into broad groups related to aspects of phage structure and replication (Fig. 6b). Conserved domains not detected in phage sequences were categorized as non-phage. Significant differences between functional profiles for PGSR phage and non-phage sequence sets (both PGSR phage and all non-phage; Fig. 6) were assessed using the *χ*^2^-test.

### Analysis of shotgun metaproteomes from human faecal microbes

Microbial cells recovered by Nycodenz extraction from stool samples (see *Recovery of bacterial cells from stool*) were suspended in 6  M guanidine isothiocyanate per 10  mM dithiothreitol/50 mM Tris pH 6.8 and processed for 4 × 30 s in a Fastprep FP120 cell disrupter (Thermo Fisher Scientific) to lyse cells and denature proteins. The guanidine isothiocyanate concentration was diluted to 1 M with 50 mM Tris (pH 6.8) and the complex sample fractionated by SDS–PAGE (12.5% gel). Protein bands were visualized by staining with colloidal Coomassie and post-separation each gel lane was divided into 28 equally sized slices (essentially as described by Schirle *et al.*^53^) and subjected to trypsin in-gel digestion according to the method of Schevchenko *et al.*^54^ The supernatant from the digested samples was removed and acidified to 0.1% TFA, dried down and reconstituted in 0.1% TFA before LC MS/MS analysis. Tryptic peptides were fractionated on a 250 × 0.075 mm^2^ reverse phase column (Acclaim PepMap100, C18, Dionex) using an Ultimate U3000 nano-LC system (Dionex) and a 2-h linear gradient from 95% solvent A (0.1% formic acid in water) and 5% B (0.1% formic acid in 95% acetonitrile) to 50% B at a flow rate of 250 nl min^−1^. Eluting peptides were directly analysed by tandem mass spectrometry using a LTQ Orbitrap XL hybrid FTMS (ThermoScientific). Derived MS/MS data (using a combined data set comprising total spectra derived from each of the 28 samples per cell pellet) were searched against databases generated from translated amino-acid sequences from all ORFs predicted in recovered PGSR contigs (*n*=2,918 ORFs for PGSR phage; *n*=6,168 ORFs for PGSR non-phage), and all contigs from human gut VLP viral metagenome assemblies^11^ (*n*=16,055 ORFs). Searches were conducted using Sequest version SRF v5 as implemented in Bioworks v3.3.1 (Thermo Fisher Scientific), assuming carboxyamidomethylation (Cys), deamidation (Asn) and oxidation (Met) as variable modifications, and using a peptide tolerance of 10 p.p.m. and a fragment ion tolerance of 0.8 Da. Filtering criteria used for positive protein identifications were Xcorr values greater than 1.5 for +1 spectra, 2 for +2 spectra and 2.5 for +3 spectra and a delta correlation (DCn) cutoff of 0.1, with a minimum of two tryptic peptides required per protein.

### Functionality of PGSR phage-encoded β-lactamases

Nucleotide sequences of PGSR phage encoding putative β-lactamase genes (Supplementary Table S4) were aligned using ClustalW^55^, and regions of homology flanking β-lactamase ORFs in all sequences were identified. Primers targeting these flanking regions were designed using Primer3 ( http://frodo.wi.mit.edu). The resulting primers (BLF 5′-TTACGGGAGGTATGGACTGC-3′; BLR 5′-TGGTTAAGCCCCTTGAACTG-3′) were used to amplify PGSR phage β-lactamase genes from total gut metagenomic DNA (See *Extraction of metagenomic DNA*). PCR amplicons were subsequently purified using the QIAquick Gel Extraction Kit (Qiagen Inc, UK), cloned into pPCR Script-Cam (Agilent, UK), and constructs transformed into *E. coli* XL10 gold. Resultant transformants were tested for their ability to grow in the presence of a range of β-lactam antibiotics (mecillinam 10 μg; ampicillin 25 μg, amoxicillin 25 μg, ceftazidime 30 μg) by disc diffusion assays conducted according to BSAC guidelines ( http://bsac.org.uk/susceptibility/). Presence of PGSR phage-derived β-lactamases in transformants conferring resistance was confirmed by direct sequencing of cloned amplicons using standard M13 primers, at GATC Sequencing Services, UK.

### Inter-individual variation in *Bacteroidales*-like phage carriage

The representation of sequences homologous to PGSR phage in gut metagenome assemblies was estimated by calculating relative abundance, based on Blast searches, as described previously by Jones *et al.*^48^,^49^ PGSR phage sequences were used to search complete gut metagenomes using Blastn (assembled data sets containing all contigs regardless of length), for contigs with high levels of similarity. Hits exhibiting a minimum of 80% identity over at least 50% of the subject sequence, and an *e*-value of 1e^−5^ or lower were considered valid, and used to calculate relative abundance (expressed as hits per Mb DNA). Subject sequence coverage thresholds were selected to minimize contribution from sequences with only limited regions of homology to PGSR phage, which are unlikely to be closely related. For the purposes of this analysis, PGSR phage contigs designated as part of the same scaffold (*n*=12) were treated as single-phage sequences and combined relative abundance calculated. To explore the potential existence of viral-enterotypes in gut microbiomes, individuals were progressively grouped according to relative abundance profiles of PGSR phage homologues, using a simple hierarchical heuristic. Starting with a randomly selected individual metagenome, individuals exhibiting similar profiles (regardless of levels of relative abundance) were assigned as ‘viral-enterotype A’, and the remainder of individuals assigned to subsequent groups in the same way until no further groupings could be made (UC). This process was repeated a second time to refine initial groupings beginning with the first individual in ‘group A’ and progressing to group D. PGSR sequences generating hits in 40% or greater of human gut metagenomes, representing the most broadly distributed phage (*n*=10), were treated as noise, and not considered during the heuristic ranking process. The existence of putative viral-enterotypes were also explored using non-metric MDS of a Bray–Curtis similarity matrix of relative abundance (hits per Mb DNA) of all PGSR sequences within each individual (including those PGSR phage sequences with homologues in 40% or more individual metagenomes and excluded from the heuristic ranking). Putative viral enterotype groupings (A, B, C, D and UC) generated from the hierarchical heuristic model were superimposed onto the MDS configuration of similarities plot and ANOSIM analysis conducted to test strength and significance of groupings (*P*<0.05; *R* statistic indicates increasing separation of groups as values approach 1). MDS and ANOSIM analysis was conducted using Primer v6 software^56^. Hierarchical heuristic ranking was carried out in Microsoft Excel.

### Construction of Emergent Self-Organizing Maps (ESOM)

For broader analysis of PGSR sequence taxonomy based on tetranucleotide useage profiles (TUPs), sequences were compared with an extended collection of bacterial chromosomes (*n*=1,700) from a wide range of habitats, as well as all phage sequences used to construct phylograms (647 phage genomes and 188 large contigs from gut viromes) (Supplementary Table S1, Supplementary Data 3–6). Relationships between sequences in this data set based on TUPs were visualized by the construction of emergent self-organizing maps using the Databionics ESOM analyser^57^ ( http://databionic-esom.sourceforge.net). Tetranucleotide frequencies transformed by *Z*-score were used with the online training algorithm over 20 training epochs, with permutation of data on each training run. Maps were generated using the correlation data distance in torroidal 2D (borderless) form and the following default training parameters: Standard bestmatch (bm) search method, a local bm search radius of 8, Gaussian weight initialization and neighbourhood kernel function, linear cooling strategy for training (radius of 24 to 1), and linear strategy for learning rate (0.5–0.1). Maps were visualized using the UMatrix background with 128 colors and height cutoff (clip) of 65%.

### Recovery of bacterial cells from stool

Microbial cells were extracted from faecal material obtained from a healthy 26-year-old male volunteer (sample collection was approved by the Clinical Research Ethics Committee of the Cork Teaching Hospitals) as described previously^58^. In summary, 10 g of stool sample was thoroughly homogenized in 20 ml phosphate buffered saline (PBS), centrifuged at 1,000 *g* for 5 min at 4 °C to pellet debris and the resulting supernatant removed to a fresh sterile tube. The faecal pellet was then washed gently three times with a single 5 ml PBS aliquot and pooled with the recovered supernatant. To separate bacterial cells from faeces, 15 ml aliquots of resulting homogenized faecal slurry were layered onto a 9.75 ml cushion of Nycodenz solution (Axis-Shield, Oslo, Norway) at a density of 1.3 g ml^−1^ Tris EDTA solution (TE buffer; 10 mM Tris, 1 mM EDTA, pH 8). Bacterial cells were harvested by centrifugation at 10,000 *g* for 6 min at 4 °C and pooled, and stored as 10% glycerol stocks in 1 ml volumes at −80 °C until required.

### Extraction of metagenomic DNA

Stocks of Nycodenz recovered cells (see *Recovery of bacterial cells from stool*) were thawed slowly on ice and 1 ml aliquots were centrifuged at 17,000 *g* for 1 min and then washed 3 × in PBS. To lyse cells, pellets were resuspended in 900 μl of TE buffer pH 8, 500 μl lysosyme (Sigma, UK; 50 mg ml^−1^ TE, pH 8), 100 μl Mutanolysin (Sigma, UK; 1 mg ml^−1^) and incubated at 37 °C for 1 h with occasional inversion. To further enhance lysis, 200 μl Proteinase K (Sigma, UK; >800 units per ml) was added to the bacterial cells and incubated at 55 °C for 1 h. Supernatant was discarded and 800 μl of 2.5% *N*-Lauryl Sarcosine solution (Sigma, UK) was added to the cells and incubated for a further 15 min at 68 °C. Following lysis, proteins were precipitated by addition of 500 μl saturated ammonium acetate solution (Sigma, UK) for 1 h at room temperature. To extract DNA an equal volume of Chloroform (Thermo Fisher Scientific UK) was added, centrifuged at 12,000 *g* for 3 min and resulting extracts removed to a fresh tube and then repeated. Resulting DNA was precipitated with ice cold ethanol (absolute; Thermo Fisher Scientific) and dissolved in sterile nuclease free water (Cambio, UK), and stored at −20 °C until use.

## Author contributions

B.V.J. and L.A.O. conceived the study. All authors contributed to study design. B.V.J., L.A.O., L.D.B., C.D., E.C. and J.C. conducted the study and analysed the data. B.V.J. and L.A.O. wrote the manuscript and all authors edited the manuscript.

## Additional information

**How to cite this article:** Ogilvie, L. A. *et al.* Genome signature-based dissection of human gut metagenomes to extract subliminal viral sequences. *Nat. Commun.* 4:2420 doi: 10.1038/ncomms3420 (2013).

## Supplementary Material

Supplementary Figures, Tables and References Supplementary Figures S1-S5, Supplementary Tables S1-S4 and Supplementary References

Supplementary Data 1 Sequences recovered from gut metagenomes using the PGSR and categorised by functional profiling.

Supplementary Data 2 Confirmation of TUP-based PGSR phage-host affiliation by alignments to bacterial chromosomes.

Supplementary Data 3 Gut associated chromosomal sequences.

Supplementary Data 4 All chromosomal sequences utilised.

Supplementary Data 5 All phage genome sequences.

Supplementary Data 6 Select sub-set of phage genome sequences.

## Figures and Tables

**Table 1:** Origin and phylogeny of driver sequences used in PGSR-based analysis of human gut metagenomes.

Driver sequence name*	**Host**	**Comments/source**	**Citations**
Phage B124–14 (accession no: HE608841)	*Bacteroides fragilis* GB-124 and closely related strains	Indicated as human gut specific	13,44
Phage B40–8 (accession no: FJ008913.1)	*Bacteroides fragilis* HSP40	Indicated as human gut specific	59,60
F2-X000044	Unconfirmed—predicted *Bacteroides*.Closely related to B124–14 and B40–8 by:Large subunit terminase gene phylogeny (Supplementary Fig. S1)Tetranucleotide profile (Supplementary Fig. S2)Gene architecture (Supplementary Fig. S3)	Recovered from Japanese human gut metagenomes by terminase gene homology	13,28
Scaffold19676_1_MH0058Scaffold70287_3_V1.UC-8Scaffold89938_1_MH0059	Unconfirmed—predicted *Bacteroides*.Closely related to B124–14 and B40–8 by:Large subunit terminase gene phylogeny (Supplementary Fig. S1)Tetranucleotide profile (Supplementary Fig. S2)Gene architecture (Supplementary Fig. S3)	Recovered from MetaHIT human gut metagenomes by terminase gene homology	13,21

PGSR, phage genome signature-based recovery.

^*^For driver sequences recovered from human gut metagenomes in previous analyses^13^, nomenclature relates directly to sequence/contig designation within metagenomes of origin. See Supplementary Figs S1–S3 for further information on driver sequences.

## References

[b1] Suttle C. A. Viruses in the sea. Nature 437, 356–361 (2005). 16163346 10.1038/nature04160

[b2] Wommack K. E. & Colwell R. R. Virioplankton: viruses in aquatic ecosystems. Microbiol. Mol. Biol. Rev. 64, 69–114 (2000). 10704475 10.1128/mmbr.64.1.69-114.2000 PMC98987

[b3] Reyes A., Semenkovich N. P., Whiteson K., Rohwer F. & Gordon J. I. Going viral: next generation sequencing applied to phage populations in the human gut. Nat. Rev. Microbiol. 10, 607–617 (2012). 22864264 10.1038/nrmicro2853 PMC3596094

[b4] Fuhrman J. A. Marine viruses and their biogeochemical and ecological effects. Nature 399, 541–548 (1999). 10376593 10.1038/21119

[b5] Brüssow H., Canchaya C. & Hardt W.-D. Phages and the evolution of bacterial pathogens: from genomic rearrangements to lysogenic conversion. Microbiol. Mol. Biol. Rev. 68, 560–602 (2004). 15353570 10.1128/MMBR.68.3.560-602.2004 PMC515249

[b6] Breitbart M. *et al.* Metagenomic analyses of an uncultured viral community from human feces. J. Bacteriol. 185, 6220–6223 (2003). 14526037 10.1128/JB.185.20.6220-6223.2003 PMC225035

[b7] Minot S. *et al.* The human gut virome: inter-individual variation and dynamic response to diet. Genome Res. 21, 1616–1625 (2011). 21880779 10.1101/gr.122705.111 PMC3202279

[b8] Stern A., Mick E., Tirosh I., Sagy O. & Sorek R. CRISPR targeting reveals a reservoir of common phages associated with the human gut microbiome. Genome Res. 22, 1985–1994 (2012). 22732228 10.1101/gr.138297.112 PMC3460193

[b9] Williamson S. J. *et al.* The Sorcerer II Global Ocean Sampling Expedition: metagenomic characterization of viruses within aquatic microbial samples. PLoS One 3, e1456 (2008). 18213365 10.1371/journal.pone.0001456 PMC2186209

[b10] Angly F. E. *et al.* The marine viromes of four oceanic regions. PLoS Biol. 4, e368 (2006). 17090214 10.1371/journal.pbio.0040368 PMC1634881

[b11] Reyes A. *et al.* Viruses in the faecal microbiota of monozygotic twins and their mothers. Nature 466, 334–338 (2010). 20631792 10.1038/nature09199 PMC2919852

[b12] Caporaso J. G., Knight R. & Kelley S. T. Host-associated and free-living phage communities differ profoundly in phylogenetic composition. PLoS One 6, e16900 (2011). 21383980 10.1371/journal.pone.0016900 PMC3044705

[b13] Ogilvie L. A. *et al.* Comparative (meta)genomic analysis and ecological profiling of human gut-specific bacteriophage ϕB124-14. PLoS One 7, e35053 (2012). 22558115 10.1371/journal.pone.0035053 PMC3338817

[b14] Lepage P. *et al.* Dysbiosis in inflammatory bowel disease: a role for bacteriophages? Gut 57, 424–425 (2008). 18268057 10.1136/gut.2007.134668

[b15] Jones B. V. The human gut mobile metagenome: a metazoan perspective. Gut Microbe. 1, 415–431 (2010). 10.4161/gmic.1.6.14087 PMC3056110 21468227

[b16] Gorski A. *et al.* New insights into the possible role of bacteriophages in host defense and disease. Med. Immunol. 2, 2 (2003). 12625836 10.1186/1476-9433-2-2 PMC151275

[b17] Colomer-Lluch M., Jofre J. & Muniesa M. Antibiotic resistance genes in the bacteriophage DNA fraction of environmental samples. PLoS One 6, e17549 (2011). 21390233 10.1371/journal.pone.0017549 PMC3048399

[b18] Waldor M. K. & Mekalanos J. J. Lysogenic conversion by a filamentous phage encoding cholera toxin. Science 272, 1910–1914 (1996). 8658163 10.1126/science.272.5270.1910

[b19] Rohwer F., Prangishvili D. & Lindell D. Roles of viruses in the environment. Environ. Microbiol. 11, 2771–2774 (2009). 19878268 10.1111/j.1462-2920.2009.02101.x

[b20] Thurber R. V., Haynes M., Breitbart M., Wegley L. & Rohwer F. Laboratory procedures to generate viral metagenomes. Nat. Protoc. 4, 470–483 (2009). 19300441 10.1038/nprot.2009.10

[b21] Qin J. *et al.* A human gut microbial gene catalogue established by metagenomic sequencing. Nature 464, 59–65 (2010). 20203603 10.1038/nature08821 PMC3779803

[b22] Pride D. T., Meinersmann R. J., Wassenaar T. M. & Blaser M. J. Evolutionary implications of microbial genome tetranucleotide frequency biases. Genome Res. 13, 145–158 (2003). 12566393 10.1101/gr.335003 PMC420360

[b23] Pride D. T., Wassenaar T. M., Ghose C. & Blaser M. J. Evidence of host-virus co-evolution in tetranucleotide usage patterns of bacteriophages and eukaryotic viruses. BMC Genomics 7, 8 (2006). 16417644 10.1186/1471-2164-7-8 PMC1360066

[b24] Deschavanne P., DuBow M. S. & Regeard C. The use of genomic signature distance between bacteriophages and their hosts displays evolutionary relationships and phage growth cycle determination. Virology J. 7, 163 (2010). 20637121 10.1186/1743-422X-7-163 PMC2917420

[b25] Marchler-Bauer A. *et al.* CDD: a Conserved Domain Database for the functional annotation of proteins. Nucleic Acids Res. 39, D225–D229 (2011). 21109532 10.1093/nar/gkq1189 PMC3013737

[b26] Tatusov R. L. *et al.* The COG database: an updated version includes eukaryotes. BMC Bioinformatics 4, 41 (2003). 12969510 10.1186/1471-2105-4-41 PMC222959

[b27] Leplae R., Hebrant A., Wodak S. J. & Toussaint A. ACLAME: a classification of Mobile genetic Elements. Nucleic Acids Res. 32, D45–D49 (2004). 14681355 10.1093/nar/gkh084 PMC308818

[b28] Kurokawa K. *et al.* Comparative metagenomics revealed commonly enriched gene sets in human gut microbiomes. DNA Res. 14, 169–181 (2007). 17916580 10.1093/dnares/dsm018 PMC2533590

[b29] Xu J. *et al.* Evolution of symbiotic bacteria in the distal human intestine. PLoS Biol. 5, e156 (2007). 17579514 10.1371/journal.pbio.0050156 PMC1892571

[b30] Murphy K. C. *et al.* Dam methyltransferase is required for stable lysogeny of the Shiga toxin (Stx2)-encoding bacteriophage 933W of enterohemorrhagic *Escherichia coli* O157:H7. J. Bacteriol. 190, 438–441 (2008). 17981979 10.1128/JB.01373-07 PMC2223730

[b31] Kruger D. H. & Bickle T. A. Bacteriophage survival: multiple mechanisms for avoiding the deoxyribonucleic acid restriction systems of their hosts. Microbiol. Rev. 47, 345–360 (1983). 6314109 10.1128/mr.47.3.345-360.1983 PMC281580

[b32] Groth A. C. & Calos M. P. Phage integrases: biology and applications. J. Mol. Biol. 335, 667–678 (2004). 14687564 10.1016/j.jmb.2003.09.082

[b33] Liu B. & Pop M. ARDB-Antibiotic resistance genes database. Nucleic Acids Res. 37, D443–D447 (2009). 18832362 10.1093/nar/gkn656 PMC2686595

[b34] Lund F. & Tybring L. 6-Amidinopenicillanic acids--a new group of antibiotics. Nat. N. Biol. 236, 135–137 (1972). 10.1038/newbio236135a0 4402006

[b35] Wootton M., Walsh T. R., Macfarlane L. & Howe R. A. Activity of mecillinam against *Escherichia coli* resistant to third-generation cephalosporins. J. Antimicrob. Chemother. 65, 79–81 (2010). 19915068 10.1093/jac/dkp404

[b36] Arumugam M. *et al.* Enterotypes of the human gut microbiome. Nature 473, 174–180 (2011). 21508958 10.1038/nature09944 PMC3728647

[b37] Dick G. J. *et al.* Community-wide analysis of microbial genome sequence signatures. Genome Biol. 10, R85 (2009). 19698104 10.1186/gb-2009-10-8-r85 PMC2745766

[b38] Duhaime M. B., Wichels A., Waldmann J., Teeling H. & Glöckner F. O. Ecogenomics and genome landscapes of marine *Pseudoalteromonas* phage H105/1. ISME J. 5, 107–112 (2011). 20613791 10.1038/ismej.2010.94 PMC3105678

[b39] Saeed I., Tang S.-L. & Halgamuge S. K. Unsupervised discovery of microbial population structure within metagenomes using nucleotide base composition. Nucleic Acid Res. 40, e34 (2011). 22180538 10.1093/nar/gkr1204 PMC3300000

[b40] Teeling H., Meyerdierks A., Bauer M., Amann R. & Glöckner F. O. Application of tetranucleotide frequencies for the assignment of genomic fragments. Environ. Microbiol. 6, 938–947 (2004). 15305919 10.1111/j.1462-2920.2004.00624.x

[b41] Ghai R. *et al.* New abundant microbial groups in aquatic hypersaline environments. Sci. Rep. 1, 135 (2011). 22355652 10.1038/srep00135 PMC3216616

[b42] Pignatelli M. *et al.* Metagenomics reveals our incomplete knowledge of global diversity. Bioinformatics 24, 2124–2125 (2008). 18625611 10.1093/bioinformatics/btn355 PMC2530889

[b43] Kim S., Rahman M., Seol S. Y., Yoon S. S. & Kim J. *Pseudomonas aeruginosa* bacteriophage PA1Ø requires type IV pili for infection and shows broad bactericidal and biofilm removal activities. Appl. Environ. Microbiol. 78, 6380–6385 (2012). 22752161 10.1128/AEM.00648-12 PMC3416619

[b44] Ebdon J., Muniesa M. & Taylor H. The application of a recently isolated strain of Bacteroides (GB-124) to identify human sources of faecal pollution in a temperate river catchment. Water Res. 41, 3683–3690 (2007). 17275065 10.1016/j.watres.2006.12.020

[b45] Gill S. R. *et al.* Metagenomic analysis of the human distal gut microbiome. Science 312, 1355–1359 (2006). 16741115 10.1126/science.1124234 PMC3027896

[b46] Teeling H., Waldmann J., Lombardot T., Bauer M. & Glöckner F. O. TETRA: a web-service and a stand-alone program for the analysis and comparison of tetranucleotide usage patterns in DNA sequences. BMC Bioinformatics 5, 163 (2004). 15507136 10.1186/1471-2105-5-163 PMC529438

[b47] Aziz R. K. *et al.* The RAST Server: rapid annotations using subsystems technology. BMC Genomics 9, 75 (2008). 18261238 10.1186/1471-2164-9-75 PMC2265698

[b48] Jones B. V., Begley M., Hill C., Gahan C. G. M. & Marchesi J. R. Functional and comparative metagenomic analysis of bile salt hydrolase activity in the human gut microbiome. Proc. Natl Acad. Sci. USA 105, 13580–13585 (2008). 18757757 10.1073/pnas.0804437105 PMC2533232

[b49] Jones B. V., Sun F. & Marchesi J. R. Comparative metagenomic analysis of plasmid encoded functions in the human gut microbiome. BMC Genomics 11, 46 (2010). 20085629 10.1186/1471-2164-11-46 PMC2822762

[b50] Felsenstein J. PHYLIP (Phylogeny Inference Package) version 3.6 Distributed by the author (Department of Genome Sciences, University of Washington: Seattle, USA, (2005).

[b51] Huson D. H. & Scornavacca C. Dendroscope 3: An interactive tool for rooted phylogenetic trees and networks. Syst. Biol. 61, 1061–1067 (2012). 22780991 10.1093/sysbio/sys062

[b52] Sun S. *et al.* Community cyberinfrastructure for advanced microbial ecology research and analysis: the CAMERA resource. Nucleic Acids Res. 39, D546–D551 (2011). 21045053 10.1093/nar/gkq1102 PMC3013694

[b53] Schirle M., Heurtier M. & Kuster B. Profiling core proteomes of human cell lines by one-dimensional PAGE and liquid chromatography-tandem mass spectrometry. Mol. Cell. Proteom. 2, 1297–1305 (2003). 10.1074/mcp.M300087-MCP200 14532353

[b54] Schevchenko A., Tomas H., Havli J., Olsen J. V. & Mann M. In-gel digestion for mass spectrometric characterization of proteins and proteomes. Nat. Protoc. 1, 2856–2860 (2007). 10.1038/nprot.2006.468 17406544

[b55] Thompson J. D., Higgins D. G. & Gibson T. J. CLUSTAL W: improving the sensitivity of progressive multiple sequence alignment through sequence weighting, position-specific gap penalties and weight matrix choice. Nucleic Acids Res. 22, 4673–4680 (1994). 7984417 10.1093/nar/22.22.4673 PMC308517

[b56] Clarke K. R. & Gorley R. N. PRIMER v6: User Manual/Tutorial PRIMER-E: Plymouth, (2006).

[b57] Ultsch A. & Moerchen F. ESOM-Maps:tools for clustering, visualisation, and classification with Emergent ESOM, Technical Report Dept. of Mathematics and Computer Science University of Marburg: Germany, No. 46, (2005).

[b58] Jones B.V. & Marchesi J. R. Transposon-aided capture (TRACA) of plasmids resident in the human gut mobile metagenome. Nat. Methods 4, 55–61 (2007). 17128268 10.1038/nmeth964

[b59] Hawkins S. A., Layton A. C., Ripp S., Williams D. & Sayler G. S. Genome sequence of the *Bacteroides fragilis* phage ATCC 51477-B1. Virol. J. 5, 97 (2008). 18710568 10.1186/1743-422X-5-97 PMC2535602

[b60] Puig M., Jofre J. & Girones R. Detection of phages infecting *Bacteroides fragilis* HSP40 using a specific DNA probe. J. Virol. Methods 88, 163–173 (2000). 10960704 10.1016/s0166-0934(00)00182-8

