# Supplementary material for: Genome signature-based dissection of human gut metagenomes to extract subliminal viral sequences
Source: Nat Commun. 2013 Sep 16;4:2420. doi: 10.1038/ncomms3420 (PMC3778543; doi:10.1038/ncomms3420)
Supplement: Supplementary Figures, Tables and References — Supplementary Figures S1-S5, Supplementary Tables S1-S4 and Supplementary References [file ncomms3420-s1.pdf]

Supplementary information for Ogilvie *et al.*

“Genome signature-based dissection of human gut metagenomes to extract subliminal viral sequences”

Supplementary Figure S1: Phylogeny of driver sequences based on large-subunit terminase genes.

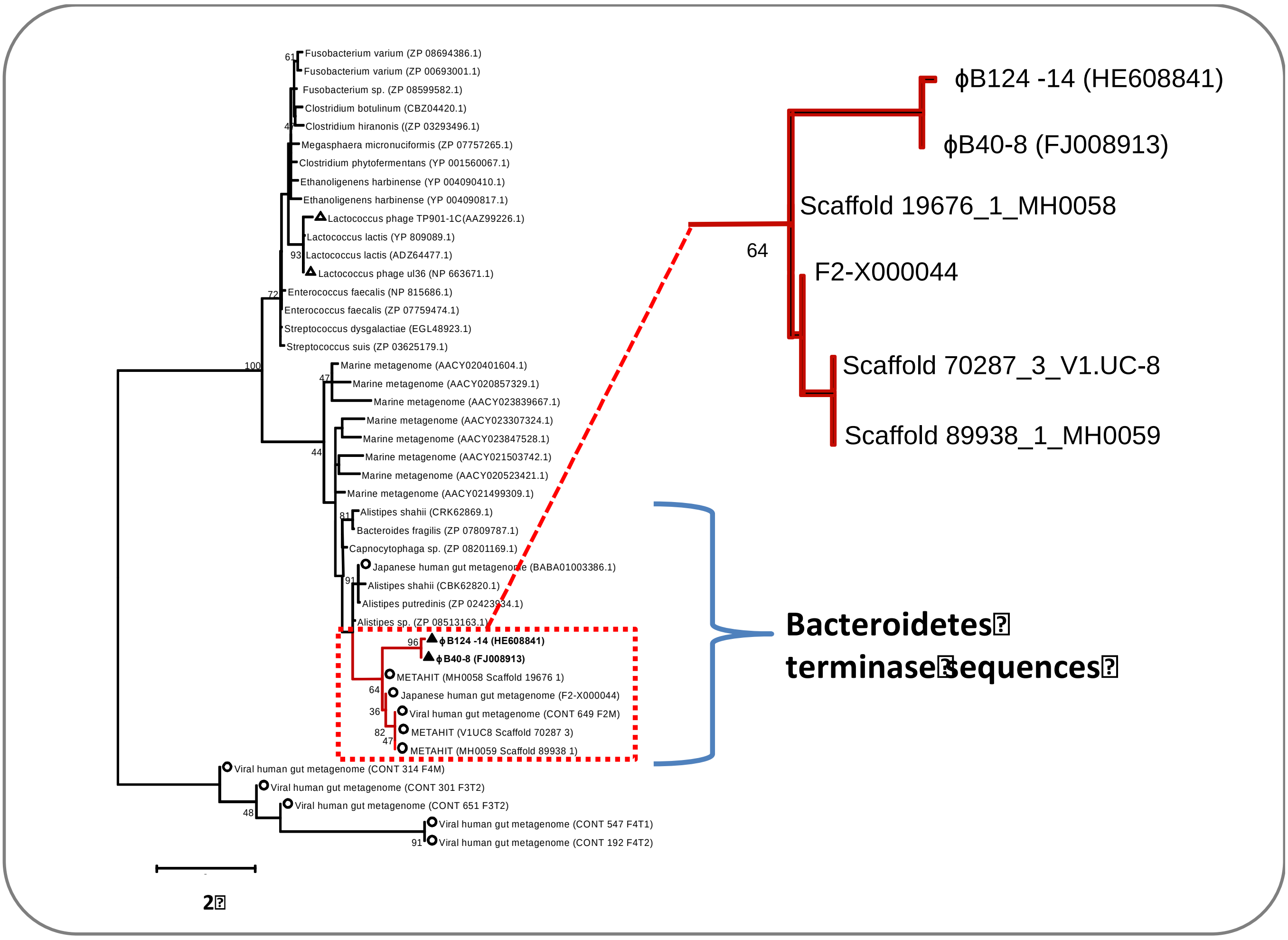

Modified from Ogilvie *et al* 2012[13]. Amino acid sequences homologous to ϕB124-14 large subunit terminase (ORF43; HE608841) were retrieved from GenBank and metagenomic datasets, including human gut microbiomes[21,28], assembled gut viromes[11], and marine microbial metagenomes[61-62]. Sequences were aligned using ClustalW[55], and unrooted consensus trees constructed using the neighbour-joining algorithm (1000 bootstrap resamplings) in MEGA v5[63]. Bootstrap values ≥40 are shown adjacent to respective tree nodes. Scale indicates amino acid substitutions. **Bracket** indicates regions populated by terminase sequences associated with members of the Phylum *Bacteroidetes*, and **branches in red** show regions populated by PGSR driver sequences (**Table 1, main text**). **Inset:** provides enlarged view of the clade populated by PGSR driver sequences. Full citations for metagenomic datasets are provided in **Supplementary Table S1**.

Supplementary Figure S2: Relationship of driver sequences to gut-associated bacterial chromosomes based on tetranucleotide usage profile.

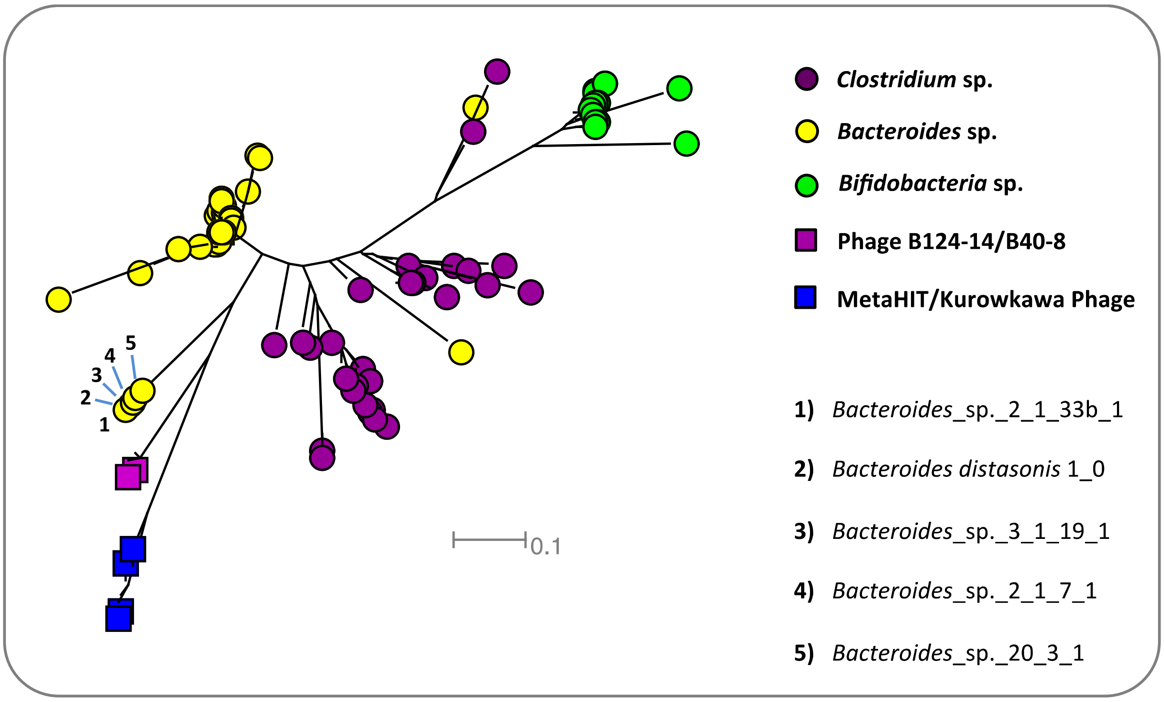

Tetranucleotide usage profiles (TUP)***** of PGSR driver sequences were compared with those present in a range of bacterial chromosomes representing common and abundant genera in the gut microbiome. Relationships were visualised by construction of phylograms using the neighbour-joining algorithm in PHYLIP 3.69[50], from distance matrices of TUP correlation scores[46]; See **Methods, main text**).

Squares: represent driver sequences used in PGSR interrogations of gut metagenomes. Blue squares denote sequences derived from either the MetaHIT gut metagenomes[21], or from Japanese gut metagenomes[28]. Purple squares represent phage genome sequences known to infect *Bacteroides fragilis*[13,59].

Circles: represent bacterial chromosomal sequences from prominent genus associated with the human gut microbiota and major phyla (*Bacteroidetes, Firmicutes, Actinobacteria*). **(1-5):** Identifies chromosomes most closely associated with phage driver sequences by TUPs, and the associated legends provide corresponding species names. Chromosomal sequences were obtained from the human gut microbiome projects and NCBI (See **Supplementary Data 3-4**).

***** TUP may be defined as the frequency of each of the 256 possible 4 nucleotide combinations (tetranucleotide) arising within a given nucleic acid sequence. TUPs in bacteriophage have previously been shown to resemble those found in the bacterial chromosomes of host bacteria, and provide an indication of host range[22-24].

Supplementary Figure S3: Physical maps of *Bacteroidales* phage sequences used as drivers in the PGSR approach.

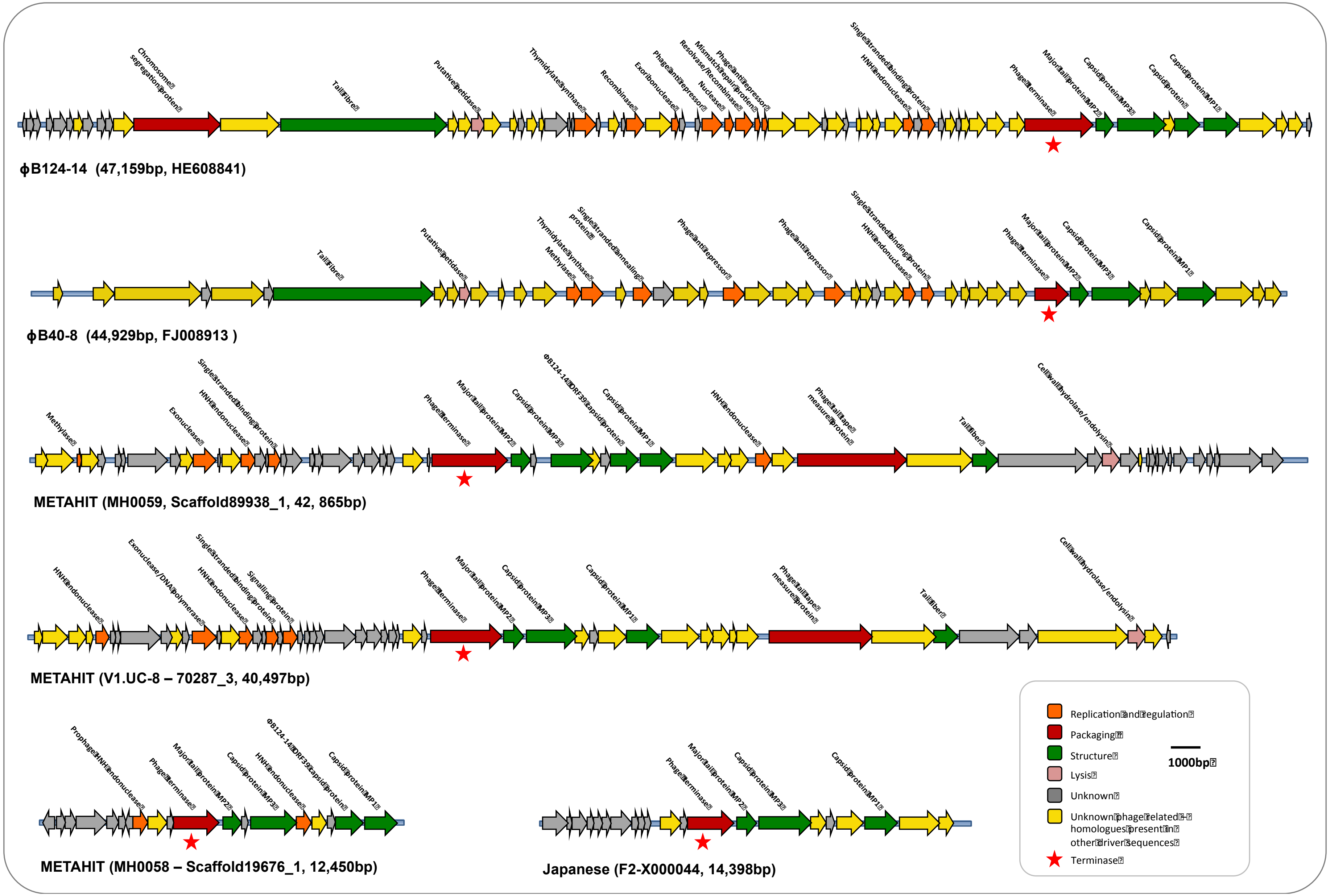

Supplementary Figure S3: ORFs encoded by *Bacteroidales*-associated phage driver sequences (**Table 1, main text**) were predicted using Glimmer V3[64], and sequence data annotated using Artemis[65]. ORF functional assignments were based on the results of BlastP, tBlastn and Conserved Domain searches as in Ogilvie *et al.*[13]. For BlastP and tBlastn only hits generating e-values of $1e^{-5}$ or lower and 20 % identity or greater were considered significant. For Conserved Domain searches only hits with an e-value of 0.01 or lower were considered significant.

Supplementary Figure S4: Visualisation of relationships between PGSR sequences, bacteriophage genomes, and bacterial chromosomes based on tetranucleotide profiles.

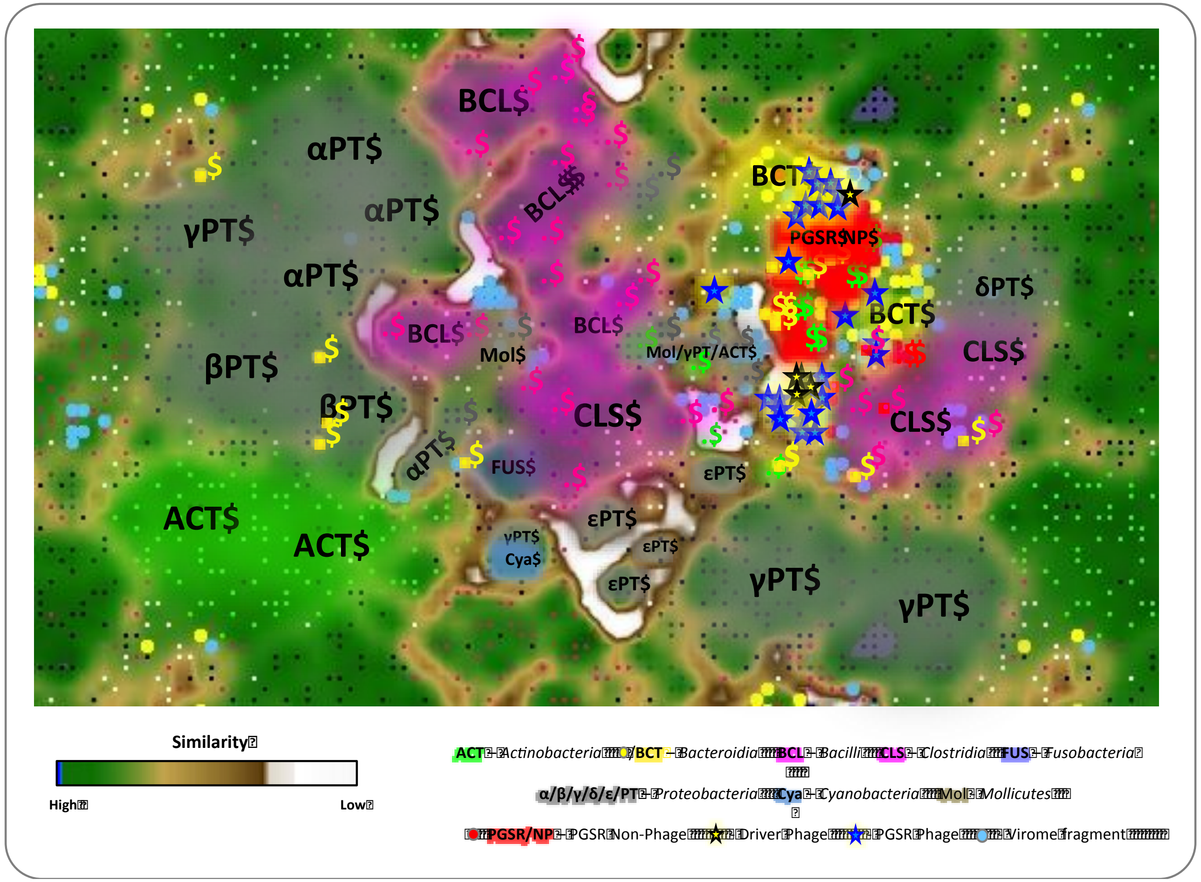

Tetranucleotide profiles were generated from 1700 bacterial chromosomal sequences, all PGSR sequences, 188 large contigs from assembled gut viromes (10 kb or over), and 647 bacteriophage genomes (also 10 kb or over), as for construction of phylograms (**Figure 4, main text**). The resulting tetranucleotide usage profiles were used to construct Emergent Self Organising Maps (ESOMs) and visualise relationships between sequences (See **Methods, main text)**. Maps represent borderless toroidal landscapes and are continuous from all edges, with the underlying topology of the ESOM "landscape" indicative of the relationship between sequences. "Valleys" (green/blue regions) describe areas populated by sequences with similar TUP profiles (high similarity), and elevated "mountain" regions (Brown/white) separate groups of less similar sequences (low similarity). Shaded areas represent regions populated predominantly (but not exclusively) by sequences from the indicated taxonomic Class, with phage sequences classified according to phylogeny of host bacteria where known or according to habitat for metagenomic sequences (gut virome and PGSR sequences). For clarity only the phylogeny of sequences belonging to bacterial classes with 40 or more representatives in the dataset are indicated by coloured regions (see figure legend). Sequences not classified are represented by white data points. For source of sequence data used see **Supplementary Table 1, and Supplementary Data 3-6.**

Supplementary Figure S5: Confirmation of functional β-lactamases encoded by *Bacteroidales*-like PGSR phage.

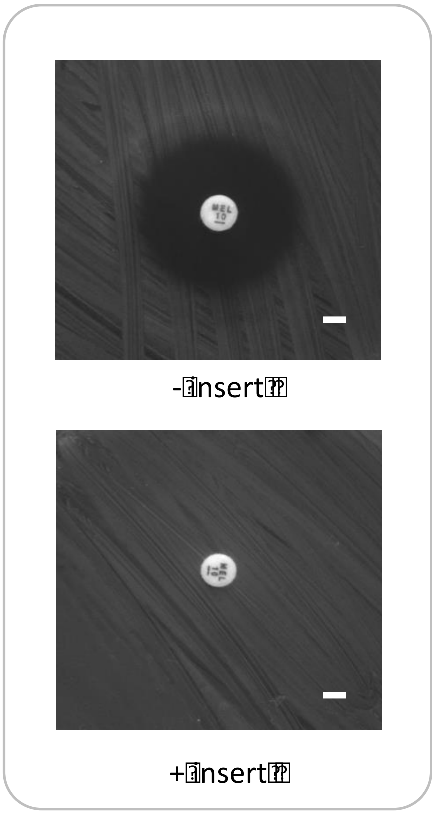

Putative β-lactamase genes encoded by PGSR phage (See **Supplementary Table S4**) were amplified from total gut metagenomic DNA and cloned in an *E. coli* host. The ability of cloned amplicons to confer resistance on surrogate *E. coli* hosts was assayed using standard disc diffusion tests with a range of β-lactam antibiotics (Amoxicillin, Mecillinam, Ampicillin, Ceftazidime). Images show the results from assays of mecillinam activity. **Upper image -** *E. coli* harboring empty vector. **Lower image -** *E. coli* harboring Type 2 PGSR phage derived β-lactamase (See **Supplementary Table S4**). Scale bars in images = 0.5 cm.

Supplementary Table S1: Overview of datasets and sequences utilised.

Dataset type	Habitat/Sequence type	Source[1]	Reference/comment
Viral Metagenomes	Human Gut **	NCBI SRA	[11]
	Swine gut	CAMERA	[66]
	Reclaimed Water	CAMERA	[67]
	Tampa Bay	CAMERA	[68]
	Sargasso Sea, Bay of British Columbia, Gulf of Mexico, Arctic Ocean	CAMERA	[10]
	Marine Virus metagenome	CAMERA	Gordon and Betty Moore Foundation Marine Microbiology Initiative. Sequenced at the Broad Institute: http://www.broadinstitute.org/annotation/viral/Phage/Home.html
	Limpolar Lake	CAMERA	[69]
	Rice Paddy Soil Viruses **	NCBI	[70]
	Marine Saltern	CAMERA	[71]
	Stromolite	CAMERA	[72]
Whole-community metagenomes	Human Gut (MetaHit) – Danish, Spanish (n=124)	EMBL	[21]; http://www.bork.embl.de/~arumugam/Qin_et_al_2010/
	Human Gut – Japanese (n=13)	CAMERA	[28]
	Human Gut – American (n=2)	CAMERA	[45]
	Canine Gut	CAMERA	[73]
	Global Ocean Sampling Expedition	CAMERA	[61-62]
	Waseca County Farm Soil	CAMERA	[74]
	Termite Gut	CAMERA	[75]
	Acid Mine Drainage	CAMERA	[76]
	Washington Lake	CAMERA	[77]
	Marine Metagenome	CAMERA	Gordon and Betty Moore Foundation Marine Microbiology Initiative. Sequenced at the Broad Institute: http://www.broadinstitute.org/annotation/viral/Phage/Home.html
	Mouse Gut	CAMERA	[78]
	Whale Fall	CAMERA	[74]
Phage genomes and bacterial chromosomes	Gut associated draft genomes; Complete and finished genome sequences from all habitats	NCBI, BI, WUGC	**NCBI:** ftp://ftp.ncbi.nih.gov/genomes/<br>Human Microbiome projects [79]:<br>**BI:** https://olive.broadinstitute.org/projects/hmp<br>**WUGC:** http://www.hmpdacc.org/reference_genomes/reference_genomes.php<br>See Supplementary Data 3-4 for further details of chromosomal datasets.
	Marine phage genomes	CAMERA	Gordon and Betty Moore Foundation Marine Microbiology Initiative. Sequenced at the Broad Institute: http://www.broadinstitute.org/annotation/viral/Phage/Home.html<br>See Supplementary Data 5-6 for further details of phage genome datasets.

	Phage genomes	NCBI	As of October 2011; http://www.ncbi.nlm.nih.gov/genomes/GenomesHome.cgi?taxid=10239 See Supplementary Data 5-6 for further details of phage genome datasets.

[1] Datasets and genome sequences utilised in this project were obtained from a range of publically accessible repositories:

CAMERA: Community Cyberinfrastructure for Advanced Microbial Ecology Research and Analysis. CAMERA Homepage**: https://portal.camera.calit2.net/gridsphere/gridsphere**. For datasets obtained as sequencing reads only, quality control was also performed using CAMERA workflows[52].

NCBI: National Centre for Biotechnology Information (Home page: http://www.ncbi.nlm.nih.gov). Phage and chromosomal sequences deposited at NCBI can be obtained from the genomes ftp indicated in the table.

NCBI SRA: Pyrosequencing reads generated from virus-like particles by Reyes *et al*.[11] were obtained from the NCBI short read archive, project SRA012183 (http://www.ncbi.nlm.nih.gov/sra).

EMBL: Metagenomes comprising the MetaHIT dataset[21] were obtained from the European Molecular Biology Laboratory database *via* the link provided in the table.

BI: The Broad Institute (http://www.broadinstitute.org/)**.** Chromosomes sequenced as part of the human microbiome project[79], and marine bacteriophage genome sequences were obtained from respective project pages as indicated in the table.

WUGC: Washington University Genome Centre (http://genome.wustl.edu): Chromosomes sequenced as part of the human microbiome project[79] were obtained from the related project homepage as indicated in the table.

****** Assembled viral metagenomes utilised for these datasets. For human gut viromes[11] both assembled and unassembled datasets were utilised in different analyses, as described in **Methods**. For assemblies of gut virome reads obtained from the NCBI SRA were processed and assembled using CAMERA workflows[52], as described in Ogilvie *et al.*[13]. For Rice Paddy Soil datasets[70] only assembled datasets were utilised, as generated by study authors.

Supplementary Table S2: Ability of alignment-based approaches to recover PGSR phage sequences.

Blast algorithm / search strategy [1]	Number of contigs recovered	Average Identity	Average Alignment length	Average Query coverage	PGSR phage sequences identified [2]
**Blastn** [a]	397	71.82% (SD = 6.9%)	560.23 nt (SD = 2273 nt)	1.45% (SD = 5.43%)	32.94%
**Megablast** [a]	24	92.91% (SD = 8.49%)	3833.33 nt (SD = 8753 nt)	11.27% (SD = 22.26%)	11.76%
**Discontiguous megablast** [a]	392	72.09% (SD = 6.90%)	432.66 nt (SD = 2273 nt)	1.22% (SD = 5.43%)	32.94%
**CRISPR spacer region alignments (Stern *et al.*[8])** [b]	991	N/A	N/A	N/A	16.47%
**tBlastn** [c] **(Capsid and Terminase)**	410	35.53% (SD = 12.77%)	295 aa (SD = 181.64 aa)	44.1% (SD = 34.21%)	22.35%

1 The ability of widely used alignment-driven approaches to identify *Bacteroidales*-like PGSR phage sequences in the MetaHIT dataset[21] were evaluated, along with recently described methods for analysis of phage sequences in metagenomes:

a Driver sequences described in **Table 1 (main text)** were used as query sequences in Blast searches of all contigs 10 kb and over in length as used in the PGSR approach. All hits generating e-values of $1e^{-3}$ or lower were recovered and rendered non-redundant by subject sequence (based on top hits by bit score – see **Methods, main paper**).

b **Data from Stern *et al.*[8].** CRISPR spacer regions identified in sequences from the MetaHIT dataset were used to search assembled contigs from these same reads, using Blastn (e-value threshold of $1e^{-4}$). Recovered sequences (10 kb in length or over) were classified as phage based on the presence of one or more phage related ORFs (**See Stern *et al.*[8] for full details).**

c Driver sequence ORFs predicted to encode capsid proteins and phage terminase sequences (**Supplementary Figure S3**) were translated and used to search MetaHIT contigs of 10 kb and over as used for PGSR analysis. All hits generating e-values of $1e^{-3}$ or lower were recovered and processed as for results from Blastn searches above (**a**).

2 Proportion of sequences recovered by the PGSR approach, and categorised as phage (PGSR phage, n = 85), also identified in alignment-driven approaches evaluated here, or CRISPR spacer based surveys by Stern *et al.* [8].

SD = Standard deviation of the mean.

Supplementary Table S3: Predicted functions of proteins encoded by PGSR sequences and gut virome fragments detected in a human gut metaproteome.

Dataset[1]	COG Class[2]	COG[2]	Function	Count[3]
**PGSR Phage & drivers** (2918 ORFs)	D	COG1196	Chromosome segregation ATPases	5
	L	COG0270	Site-specific DNA methylase	1
	L	COG4974	Site-specific recombinase XerD	1
	Unclassified	-	-	1
**Gut Virome** (16055 ORFs)	D	COG1196	Chromosome segregation ATPases	1
	R	COG1444	Predicted P-loop ATPase fused to an acetyltransferase	2
	R	COG5271	AAA ATPase containing von Willebrand factor type A (vWA) domain	2
	Unclassified	-	-	5
**PGSR Non-Phage** (6168 ORFs)	C	COG0039	Malate/lactate dehydrogenases	1
	C	COG1838	Tartrate dehydratase beta subunit/Fumarate hydratase class I, C-terminal domain	2
	C	COG1951	Tartrate dehydratase alpha subunit/Fumarate hydratase class I, N-terminal domain	2
	E	COG0136	Aspartate-semialdehyde dehydrogenase	1
	E	COG2195	Di- and tripeptidases	1
	G	COG0148	Enolase	3
	G	COG0149	Triosephosphate isomerase	1
	G	COG0191	Fructose/tagatose bisphosphate aldolase	1
	G	COG0205	6-phosphofructokinase	3
	G	COG0363	6-phosphogluconolactonase/Glucosamine-6-phosphate isomerase/deaminase	2
	G	COG4806	L-rhamnose isomerase	1
	IQR*	COG1028	Dehydrogenases with different specificities (related to short-chain alcohol dehydrogenases)	1
	J	COG0480	Translation elongation factors (GTPases)	2
	J	COG0532	Translation initiation factor 2 (IF-2; GTPase)	1
	K	COG2207	AraC-type DNA-binding domain-containing proteins	2
	KG*	COG1940	Transcriptional regulator/sugar kinase	1
	L	COG0468	RecA/RadA recombinase	1
	M	COG0481	Membrane GTPase LepA	1
	M	COG1087	UDP-glucose 4-epimerase	1
	O	COG0450	Peroxiredoxin	4
	R	COG2103	Predicted sugar phosphate isomerase	2
	R	COG5271	AAA ATPase containing von Willebrand factor type A (vWA) domain	1
	S	COG2120	Uncharacterized proteins, LmbE homologs	2
	T	COG0642	Signal transduction histidine kinase	2
	T	COG3292	Predicted periplasmic ligand-binding sensor domain	2
	TK*	COG0745	Response regulators consisting of a CheY-like receiver domain and a winged-helix DNA-binding domain	3

[1] Amino acid sequences from all predicted PGSR ORFs, as well as all contigs assembled from gut viromes[11] **(Supplementary Table S1**), were used to construct custom databases and search mass spectra (177729 spectra) derived from a shotgun metaproteome of a human faecal microbiome (see **Methods, main text).** Figures in parentheses show the total number of ORF sequences predicted in each dataset, and used in searches of mass spectra.

[2] For sequences detected in the metaproteome, putative functions were assigned based on COG searches (e-value = $1e^{-3}$ or lower). COG classes: **C** – Energy production and conversion; **D** – Cell cycle control, cell division, chromosome partitioning; **E** – Amino acid transport and metabolism; **G** – Carbohydrate transport and metabolism; **I** – Lipid transport and metabolism; **J** – Translation, ribosomal structure and biogenesis; **K** – Transcription; **L** – Replication, recombination and repair; **M** – Cell wall/membrane/envelope biogenesis; **O** – Posttranslational modification, protein turnover, chaperones; **Q** – Secondary metabolites biosynthesis, transport and catabolism; **R** – General function prediction only; **S** – Function unknown; **T** – Signal transduction mechanisms. **Unclassified** – ORFs detected in human gut but without representation in the COG database.

***** ORFs with functions assigned to multiple COG classes.

[3] Indicates the number of ORFs from each dataset detected in the gut metaproteome, and ascribed a particular function based on COG searches.

Supplementary Table S4: Homology and functionality of PGSR phage encoded ORFs containing β-lactamase conserved domains.

β-lactamase type	Sequence Title[1]	Proven function	BlastP top hit[2]
**Type 1**	scaffold5177_1_MH0014 (healthy)<br>scaffold739_5_MH0013 (healthy)<br>scaffold8327_6_MH0085 (healthy)	No	Metallo-beta-lactamase domain-containing protein; *Bacteroides* sp.D22 and *Bacteroides* sp. 1_1_30. 70% (169/240) identity, $2e^{-220}$
**Type 2**	scaffold262_1_MH0071 (healthy)<br>scaffold18411_2_MH0020 (healthy)<br>scaffold4201_1_MH0085 (healthy)<br>scaffold11813_5_MH0082 (healthy)<br>scaffold38994_2_MH0042 (healthy)	**Yes ***	Hypothetical protein; *Bacteroides stercoris* BACSTE_02214. 100% (241/241) identity, $1e^{-178}$
**Type 3**	scaffold17211_1_MH0068 (healthy)<br>scaffold17094_1_O2.UC-19 (IBD)	No	Metallo-beta-lactamase domain-containing protein; *Bacteroides* sp.D22 and *Bacteroides* sp. 1_1_30. 100% (242/242) identity, $1e^{-179}$
**Type 4**	scaffold17066_10_V1.UC-14 (IBD)	No	Hypothetical protein; *Bacteroides stercoris* BACSTE_02214. 99% (240/241) identity, $3e^{-177}$
**Type 5**	scaffold98597_1_V1.UC-10 (IBD)	No	Hypothetical protein; *Bacteroides stercoris* BACSTE_02214. 99% (238/241) identity, $6e^{-177}$

1 Denotes title of original sequence in MetaHit dataset[21], with details in parentheses denoting health status of individual.

2 Top hit by bit score from BlastP searches of the nr dataset using amino acid sequences from each PGSR phage β-lactamase type.

* Cloned PCR amplicon confers resistance to mecillinam in *E. coli* host (see **Supplementary Fig. S5**).

Supplementary References.

61. Rusch, D.B. *et al.* The Sorcerer II Global Ocean Sampling expedition: northwest Atlantic through eastern tropical Pacific. *PloS Biol.* **5**, e77 (2007).

62. Yoosef, S. *et al.* The Sorcerer II Global Ocean Sampling expedition: expanding the universe of protein families. *PLoS Biol.* **5**, e16 (2007).

63. Tamura, K. *et al.* MEGA5: molecular evolutionary genetics analysis using maximum likelihood, evolutionary distance, and maximum parsimony methods. *Mol Biol Evol.* **28**, 2731–2739 (2011).

64. Delcher, A.L., Harmon, D, Kasif, S., White, O., Salzberg, S.L. Improved microbial gene identification with GLIMMER. *Nucleic Acids Res.* **27**, 4636-4641 (1999).

65. Rutherford, K. *et al.* Artemis: sequence visualization and annotation. *Bioinformatics* **16**, 944-945 (2000).

66. Allen, H.K. *et al.* Antibiotics in feed induce prophages in swine fecal microbiomes. *MBio* **6**, e00260-11 (2011).

67. Rosario, K., Nilsson, C., Lim, Y.W., Ruan, Y. & Breitbart, M. Metagenomic analysis of viruses in reclaimed water. *Environ. Microbiol.* **11**, 2806-2802 (2009).

68. McDaniel, L. *et al.* Metagenomic analysis of lysogeny in Tampa Bay: implications for prophage gene expression. *PLoS ONE* **3**, e3263 (2008).

69. López-Beueno, A. *et al.* High diversity of the viral community from an Antarctic lake. *Science* **326**, 858-861 (2009).

70. Kim, K.H. *et al.* Amplification of uncultured single-stranded DNA viruses from rice paddy soil. *Appl. Environ. Microbiol.* **74**, 5975-5985 (2008).

71. Dinsdale, E.A. *et al.* Functional metagenomic profiling of nine biomes. *Nature* **452**, 629-632 (2008).

72. Desneus, C. *et al.* Biodiversity and biogeography of phages in modern stromatolites and thrombolites. *Nature* **452**, 340-343 (2008).

73. Swanson, K.A. *et al.* Phylogenetic and gene-centric metagenomics of the canine intestinal microbiome reveals similarities with humans and mice. *ISME J.* **5**, 639-649 (2011).

74. Tringe, S.G. *et al.* Comparative metagenomics of microbial communities. *Science* **308**, 554 – 557 (2005).

75. Warnecke, F. *et al.* Metagenomic and functional analysis of hindgut microbiota of a wood-feeding higher termite. *Nature* **450**, 560-565 (2007).

76. Tyson, G.W. *et al.* Community structure and metabolism through reconstruction of microbial genomes from the environment. *Nature* **428**, 37-43 (2004).

77. Kalyuzhnaya, M.G. *et al.* High-resolution metagenomics targets specific functional types in complex microbial communities. *Nat. Biotechnol.* **26**, 1029-1034 (2008).

78. Turnbaugh, P.J. *et al.* An obesity-associated gut microbiome with increased capacity for energy harvest. *Nature* **444**, 1027-1031 (2006).

79. Nelson, K. *et al.* A catalog of reference genomes from the human microbiome. *Science* **328**, 994-999 (2010).
